# The multilayer community structure of medulloblastoma

**DOI:** 10.1016/j.isci.2021.102365

**Published:** 2021-03-26

**Authors:** Iker Núñez-Carpintero, Marianyela Petrizzelli, Andrei Zinovyev, Davide Cirillo, Alfonso Valencia

**Affiliations:** 1 Barcelona Supercomputing Center (BSC), C/ Jordi Girona 29, 08034, Barcelona, Spain; 2 Institut Curie, PSL Research University, 75005 Paris, France; 3 INSERM, U900, 75005 Paris, France; 4 MINES ParisTech, PSL Research University, CBIO-Centre for Computational Biology, 75006 Paris, France; 5 Lobachevsky University, 603000 Nizhny Novgorod, Russia; 6 ICREA - Institució Catalana de Recerca i Estudis Avançats, Pg. Lluís Companys 23, 08010, Barcelona, Spain

**Keywords:** Proteomics, Cancer Systems Biology, Cancer

## Abstract

Multilayer networks allow interpreting the molecular basis of diseases, which is particularly challenging in rare diseases where the number of cases is small compared with the size of the associated multi-omics datasets. In this work, we develop a dimensionality reduction methodology to identify the minimal set of genes that characterize disease subgroups based on their persistent association in multilayer network communities. We use this approach to the study of medulloblastoma, a childhood brain tumor, using proteogenomic data. Our approach is able to recapitulate known medulloblastoma subgroups (accuracy >94%) and provide a clear characterization of gene associations, with the downstream implications for diagnosis and therapeutic interventions. We verified the general applicability of our method on an independent medulloblastoma dataset (accuracy >98%). This approach opens the door to a new generation of multilayer network-based methods able to overcome the specific dimensionality limitations of rare disease datasets.

## Highlights

• The molecular interpretation of rare diseases is a challenging task
• Multilayer networks allow patient stratification and explainability
• We identify subgroup-specific genes and multilayer associations in medulloblastoma
• Multilayer community analysis enables the molecular interpretation of rare diseases

## Graphical abstract

**Figure:**
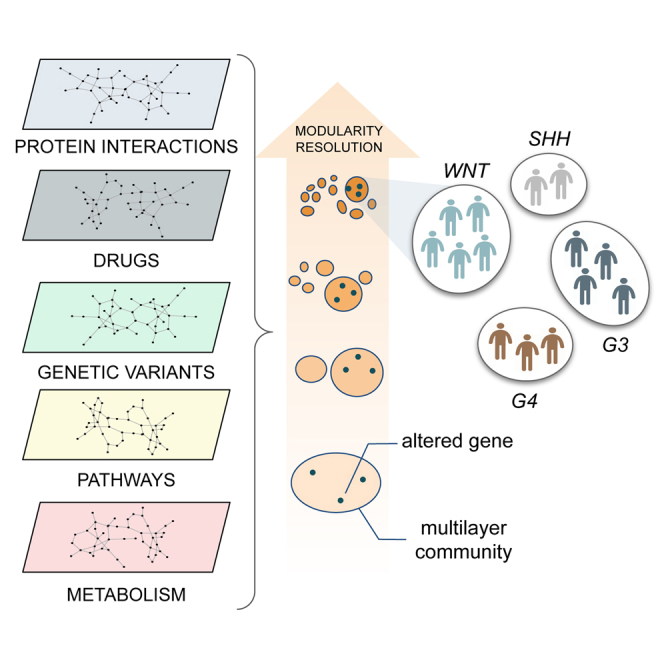

## Introduction

To improve our understanding of complex systems, it is crucial to take into account the multiple types of relationships that inherently define natural systems. The study of the so-called multilayer networks (alternatively multiplex networks) has recently become one of the most important directions in network science (Kivela et al., 2014; Aleta and Moreno 2019). A multilayer network is a network organized into multiple layers representing different types of nodes and edges (Figure S1). Despite offering the means to achieve a comprehensive view of human diseases by accounting for the complexity of accumulated biomedical data, biological multilayer networks exhibit a range of research challenges that still require substantial investigation (Kristensen et al., 2014). Among them, community detection in multilayer networks is an area of investigation that is particularly promising for biomedicine, facilitating the evaluation of relevant associations among genes and the identification of candidate targets for drug development and repurposing (Halu et al., 2019; Valdeolivas et al., 2019).

Popular strategies for community detection in networks include the Louvain algorithm (Blondel et al., 2008), a greedy optimization technique, to maximize a network structural metric that is called modularity (Newman and Girvan 2004). Modularity is defined as the fraction of edges within a group of nodes that is significantly enriched when compared with a random model. It measures the strength of a given partition of the network (Reichardt and Bornholdt 2006). The Louvain algorithm is one of the most widely used meta-heuristics for community detection in large networks. It outperforms other community detection algorithms in accuracy, scalability, and computing time (Yang et al., 2016). Moreover, the algorithm is implemented in a number of network analysis software, and it has been recently adapted to multilayer networks (Didier et al. 2015; Didier et al. 2018).

Nevertheless, community structure determination in networks remains an open problem to such an extent that the preferred formulation of communities is often domain specific (Porter et al. 2009). One major conundrum of modularity-based approaches to community detection is the intrinsic limit of resolution, by which it is *a priori* impossible to rule out that a community defined at a certain level of resolution may be composed of a cluster of smaller communities (Fortunato and Barthélemy 2007; Lancichinetti and Fortunato 2011). In other words, multiple topological descriptions, each one with its own importance, coexist at different scales that are detected at alternative values of resolution (Arenas et al. 2008). As a consequence, the identification of meaningful network communities, such as groups of genes of interest that robustly express strong associations, heavily depends on the choice of the resolution value to be used. This limitation can be overcome through the identification of stable partitions at different values of resolution. Indeed, the detection of persistent partitions when changing the resolution is indicative of strong modular structures (Arenas et al. 2008).

**Figure 1:**
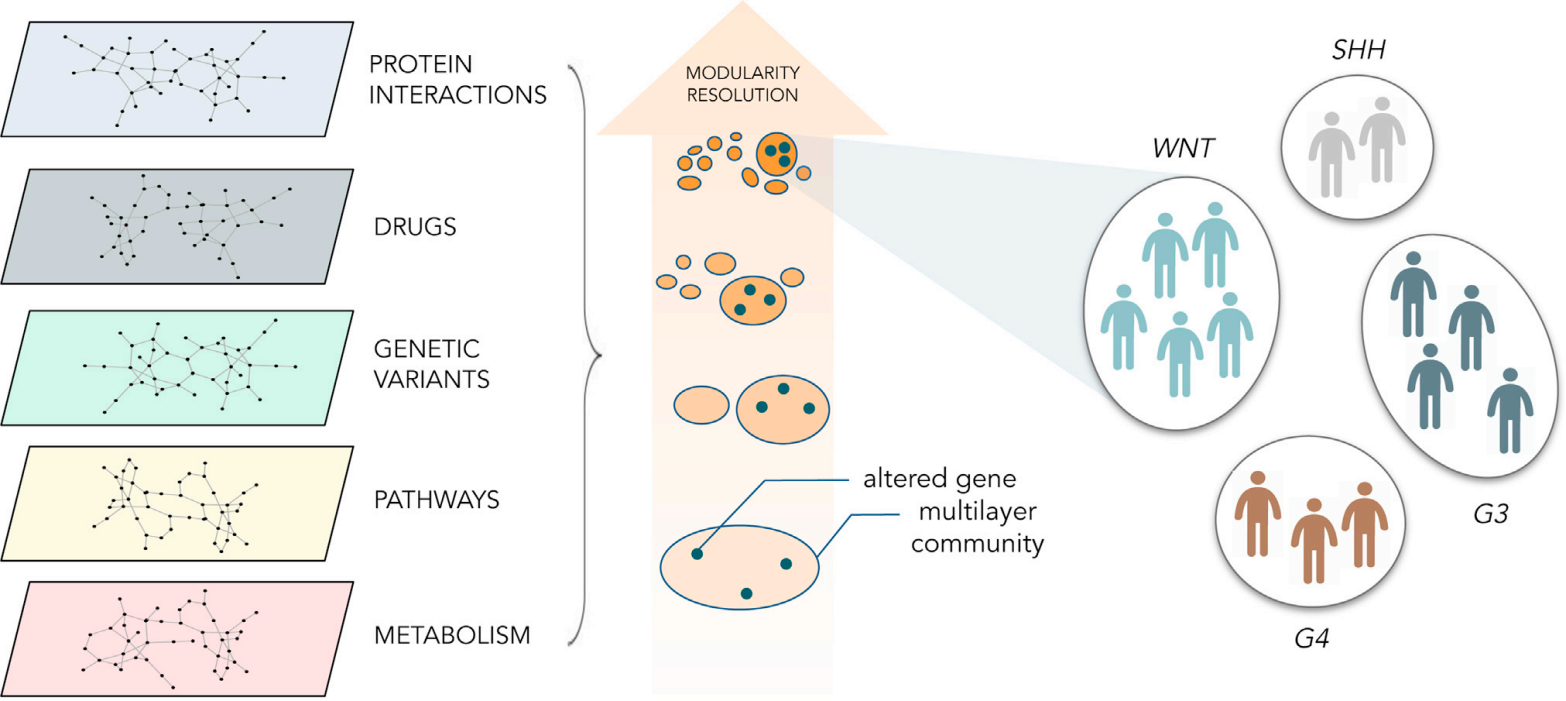
Multilayer community structure analysis of medulloblastoma subgroups Using multilayer community structure analysis on a network describing gene-gene associations based on protein interactions, drug targets, genetic variants, pathways, and metabolic reactions, we identified the minimum sets of altered genes that optimally cluster the patients with medulloblastoma into previously described subgroups. See also Figures S1 and S2.

**Figure 2:**
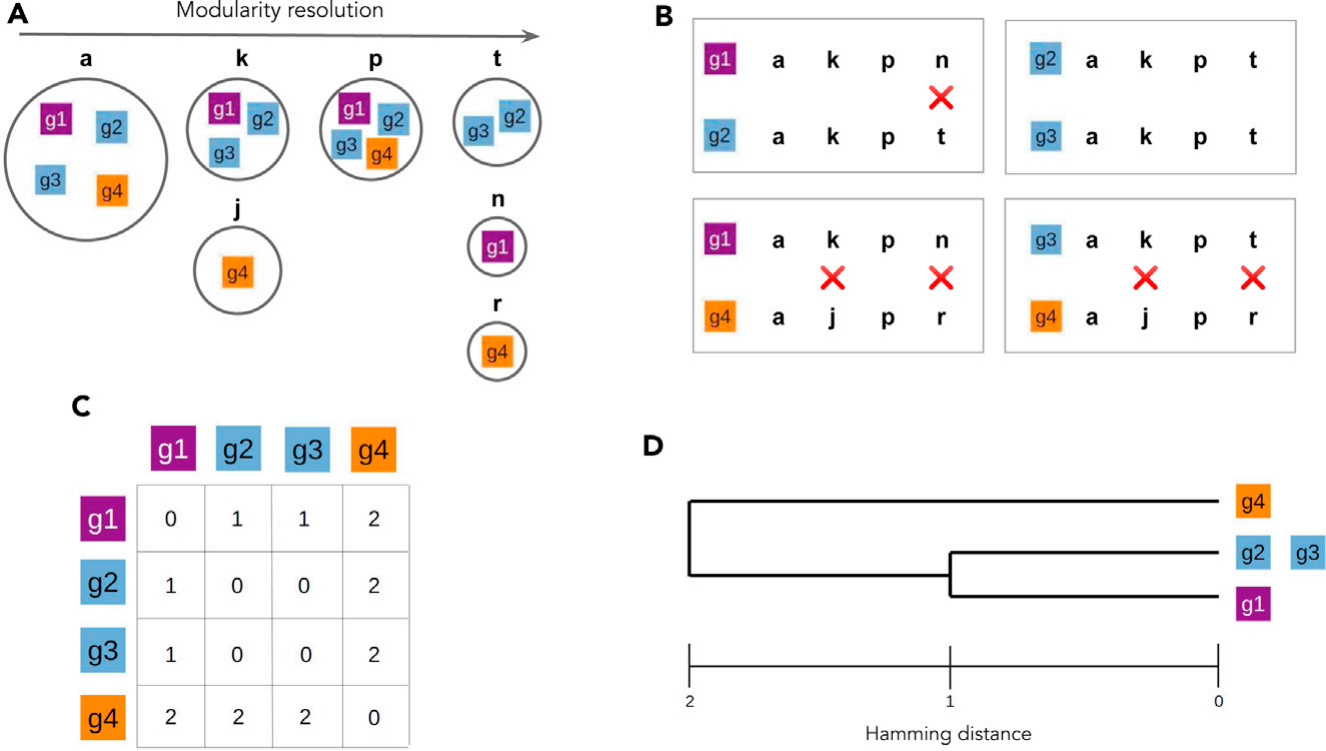
Identification of multilayer community trajectories (A–D) For a given set of genes, we identified the multilayer communities to which they belong in a range of modularity resolution (A). We then computed the pairwise Hamming distances of the trajectories of communities visited by each gene (B). The corresponding distance matrix (C) was represented in the form of a dendrogram (D) used for clustering analysis. See also Figure S3.

Here we implemented a methodology to identify groups of genes that are systematically found to belong to the same communities across a range of different resolution values. In this view, two or more genes of interest that are consistently found in the same communities at different values of resolution will be deemed strongly associated based on the multiple biological evidence from the multilayer network. We applied this concept to the analysis of the multilayer community structure of genes altered in a cohort of patients with medulloblastoma (MB) who were previously stratified based on proteogenomic data (Forget et al., 2018) (Figure 1). To this aim, we implemented a dimensionality reduction methodology based on the persistent association of genes in the multilayer network communities (see methods: “multilayer community structure analysis” and Figure 2).

MB is a malignant and fast-growing primary central nervous system tumor, which originates from embryonic cells of the brain or spinal cord with no known causes and a preferential manifestation in children (aged 1–9 years). Despite being rare, MB is the most common cancerous brain tumor in children. Four molecular disease subgroups of pediatric MB with distinct clinicopathological features have been identified: WNT, SHH, Group 3 (G3), and Group 4 (G4) (Taylor et al., 2012; Northcott et al., 2011). WNT is associated with the most favorable prognosis, whereas SHH and G4 are associated with intermediate-level prognosis and G3 with the worst outcome. Seven genes exhibit recurrent genetic alterations in the four subgroups (*SHH* in SHH group, *CTNNB1* in WNT group, *MYC* and MYCN in G3 and G4, *ERBB4*, *SRC,* and *CDK6* in G4 (Kool et al., 2012; Ramaswamy et al., 2016; Taylor et al., 2012; Northcott et al., 2014; Robinson et al., 2012; Northcott et al., 2017; Kool et al., 2014; Clifford et al., 2006; Forget et al., 2018). Each subgroup presents substantial biological heterogeneity and survival differences (Jones et al., 2012) so much so that the identification of more than four subgroups has been recently proposed, in particular as concerns the heterogeneity of G3 and G4 (Schwalbe et al., 2017).

Our results show that our multilayer community structure analysis is able to recapitulate the four MB subgroups (accuracy 94.94%), as well as better characterize them by identifying distinct minimal sets of genes with strong associations based on multiple layers of evidence (Figure S2). We further verify the applicability of our method using an independent MB multi-omics dataset, achieving a very high performance also in this case (accuracy 98.29%). This work represents an important step forward not only in the characterization of MB subgroups but also, in general, in rare tumor research, where the absence of large patient sample cohorts makes the identification of supporting evidence for candidate genes an extremely challenging task.

## Results

### Multilayer community trajectories

To implement a way to monitor the behavior of multilayer communities containing MB genes upon changes of the modularity resolution, we initially sought to take into account gene mentions in abstracts of scientific publications about MB (see methods: “data sources of medulloblastoma genes”). By interrogating PubTator Central (PTC) (Wei et al., 2019), we retrieved a list of 1,941 multi-species genes, consisting of 1,475 human genes (76%), 389 murine genes (20%), and 77 genes of other species (4%). We identified the multilayer communities to which the human genes (1,387 out of 1,475, represented in the multilayer network) belong in a range of modularity resolution (see methods: “multilayer community structure analysis” and Figure S3). We conceived this particular analysis as a proof of concept for the multilayer community structure analysis.

**Figure 3:**
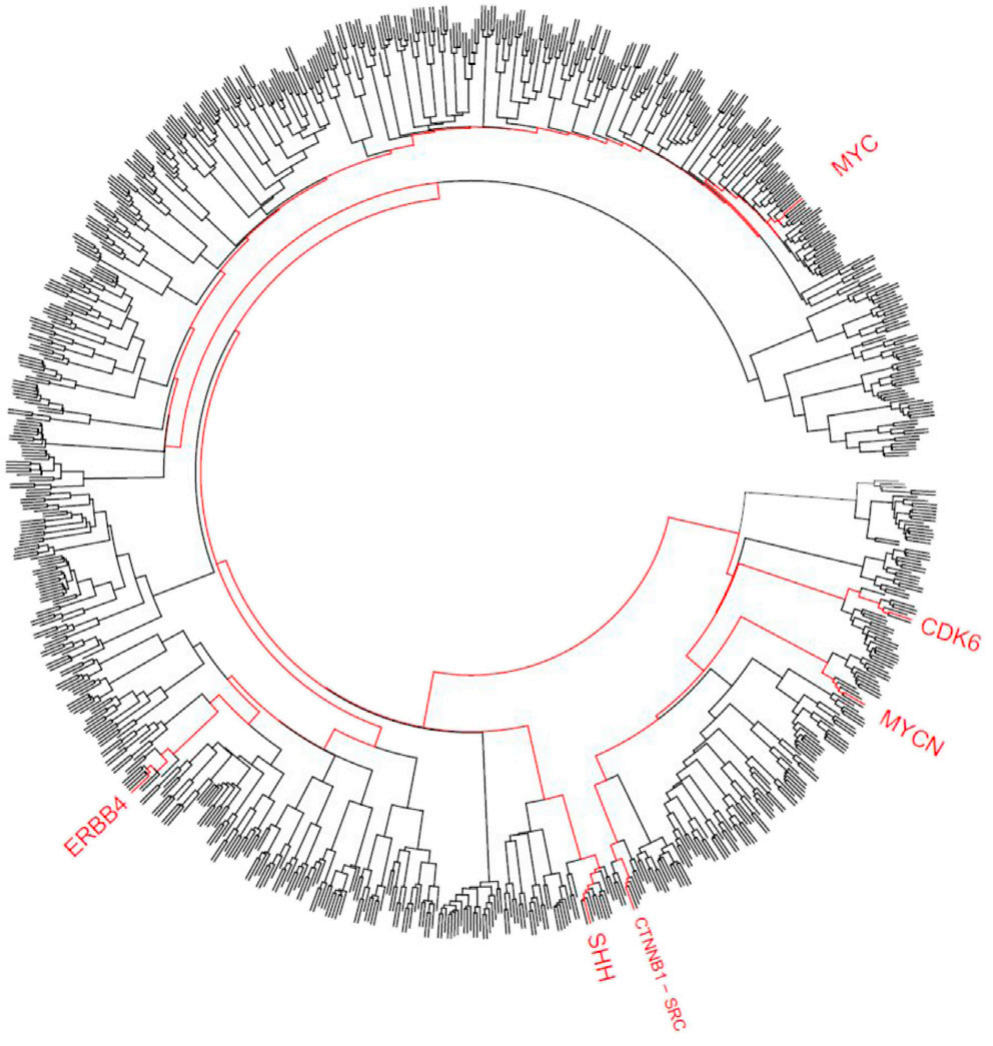
Dendrogram of multilayer community trajectories The dendrogram represents the Hamming distance among the trajectories of the communities visited by each gene associated to medulloblastoma by text mining in a range of modularity resolution (see methods: “multilayer community structure analysis”). Trajectories of seven genes that are known to characterize medulloblastoma subgroups (see introduction) are highlighted in red. See also Figure S4.

As shown in Figure 3, there are plain differences in the trajectories of the communities that are visited by each gene. Interestingly, the trajectories of seven genes, whose recurrent genetic alterations are well-known hallmark features of the four molecular disease subgroups (see introduction), branch off from well-separated communities, with the exception of *SRC* and *CTNNB1*, which are physical interactors (IntAct: EBI-15951997).

The landscape of these multilayer community trajectories can be further explored to investigate the so-called operations on dynamic communities (Cazabet et al. 2017), such as birth (a new community appears), death (a community vanishes), and resurgence (a community disappears and appears again later on). Along the explored range of modularity resolution, the 2,186 unique multilayer communities of the text-mined MB genes experience a total of 2,517 death events and 673 resurgence events (Figure S4), indicating not only a high level of instability (all communities disappear at least once) but also a high level of commutability (some communities reappear several times with the same exact composition). These observations led us to realize that each gene is characterized by its own journey throughout the communities found at different levels of resolution. For this reason, we further tested the hypothesis that tracing such trajectories for a set of disease-related genes could be exploited for patient clustering purposes (see methods: “identification of the minimal set of genes that define medulloblastoma subgroups”).

### Medulloblastoma patient stratification through multilayer structure analysis

We sought to use the trajectories of the multilayer communities visited by the genes altered in MB to achieve patient stratification. Our reference (ground truth) consists of the four classical subgroups (WNT, SHH, G3, G4), which represent a standard categorization of MB despite substantial heterogeneity and the possibility of a more granular stratification have been reported (see introduction). The four subgroups have been recently investigated via network fusion using a cohort of patients with proteogenomic information (Forget et al., 2018). We reanalyzed this cohort to optimally recapitulate the four subgroups, while aiming to reduce the number of critical genes required for this stratification.

We retrieved lists of genes altered in 35 patients who display complete datasets (DNA methylation, RNA sequencing, proteomics, and phosphoproteomics) (see methods: “data sources of medulloblastoma genes”). Partial datasets are available for three additional patients (MB10, MB21, and MB33) that we retained as a validation set (see results: “sensitivity analyses”). We performed a hierarchical clustering based on the multilayer community trajectories of an optimal selection of minimal sets of genes. Optimality means that the features of these selected genes, in terms of their representation in the multilayer communities (parameter λ) and the similarity of their trajectories (parameter θ), allow clustering patients with the maximum accuracy and Matthews correlation coefficient (MCC) to the four subgroups of reference (see methods: “identification of the minimal set of genes that define medulloblastoma subgroups”).

**Figure 4:**
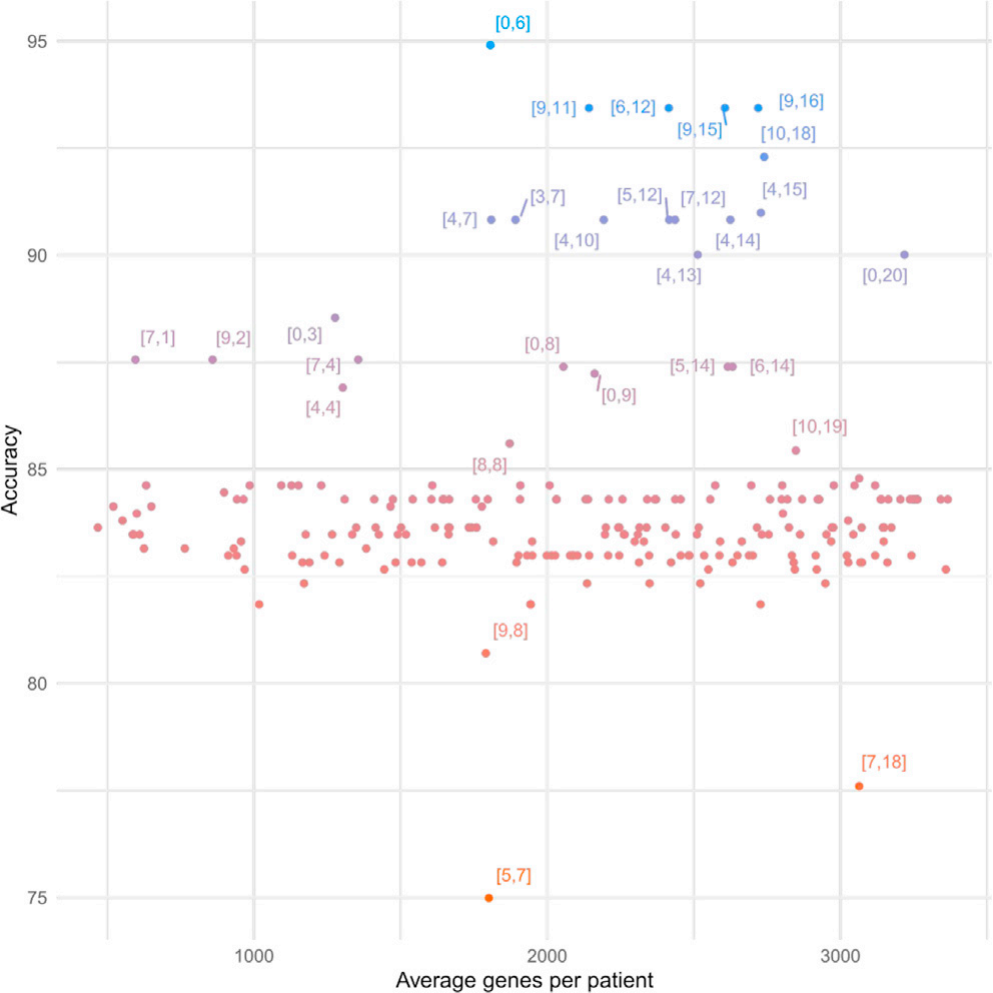
Parameters optimization Scatterplot comparing the average genes per patient obtained by each iteration of the optimization procedure (see methods: “identification of the minimal set of genes that define medulloblastoma subgroups”) and its corresponding accuracy. Values next to each point highlight the corresponding [θ,λ] combination. See also Figures S5–S7 and Tables S1–S3.

**Figure 5:**
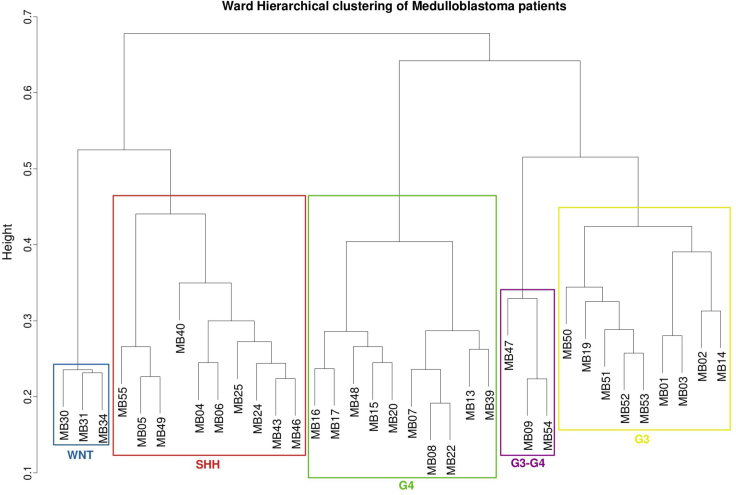
Clustering of medulloblastoma patients Ward's linkage hierarchical clustering obtained at λ = 6 and θ = 0. The rectangles indicate the five clusters suggested by PAM (partitioning around medoids) criteria. The color of each cluster indicates the original patient stratification into the four medulloblastoma subgroups (Forget et al., 2018): WNT (blue), SHH (red), Group 4 (G4, green), Group 3 (G3, yellow). A fifth cluster is depicted in purple, including three patients originally assigned to subgroups G3 (MB47) and G4 (MB09 and MB54). See also Figures S8 and S9 and Tables S4, S5, and S6.

We achieved the highest accuracy (94.94%) and MCC (87%) with five clusters (WNT, SHH, G4, G3, and G3-G4), by selecting for each patient those genes that are represented in the communities in sets of at most 6 (λ = 6) and that are always part of the same communities along their trajectories (θ = 0) (Figures 4 and 5, Tables S1–S3). Strikingly, such high accuracy corresponds to a strict selection of genes, indicating that only a small portion of the genes altered in a patient is sufficient to accomplish an accurate patient segregation. This observation implies that the selected genes are tightly associated and never leave the communities they belong to along their trajectories. An important aspect of this result is that, despite our reference being of four subgroups, we identified five clusters, indicating that only few patients escape the classical categorization and subtler stratas may exist, as suggested in recent studies (Schwalbe et al., 2017; Archer et al., 2018).

### Classification of patients with partial molecular information

As the datasets of three patients consist of partial molecular information (see methods: “data sources of medulloblastoma genes”), we excluded these samples from the parameter optimization procedure and used them as a validation set. The three patients belong to subgroups G4 (patient MB10) and WNT (patients MB21 and MB33) (Forget et al., 2018). We assigned each one of the three patients to the cluster of the most similar among the remaining 35 patients based on the Jaccard Index (J) parametrized by the optimal θ and λ (see methods: “identification of the minimal set of genes that define medulloblastoma subgroups”). Patient MB10 shows the highest similarity to patient MB22 (J = 0.263), who belongs to G4 subgroup likewise eight patients in the following ranking positions (Table S4). Patient MB21 shows the highest similarity to three patients of the WNT subgroup (MB31 J = 0.2653; MB34 J = 0.2631; MB30 J = 0.2601). Finally, patient MB33 shows high similarity to two patients of WNT subgroup (MB30 J = 0.2168; MB34 J = 0.2106). Of note, patient MB31 of the WNT subgroup is the fourth most similar patient to MB33 (J = 0.2080), MB16 of the G4 subgroup being the third (J = 0.2081). These results show that the parameters for gene selection optimized based on patients with complete molecular information allow classifying the patients who have only partial molecular information with high accuracy (all three patients are correctly classified).

### Robustness analyses

The identified values of θ and λ, optimized on 35 patients, correspond to an average of 1,812.74 genes per patient (SD = 106.97) (i.e., an average dimensionality reduction of 87.56% (SD = 0.44) per patient) (Table S5). Moreover, some of these genes are uniquely found among all patients of distinct clusters (148 genes in G3 patients; 83 genes in SHH patients; 115 genes in G4 patients; 46 genes in G3-G4 patients; 260 genes in WNT patients).

We evaluated the robustness of our results with two types of robustness analyses. In the first analysis, we shuffled the altered genes across the cohort 10,000 times, maintaining the same number of genes for each patient as in the original data. This procedure yielded an average accuracy of 54.76% (SD = 0.11) with θ = 0 and λ = 6 (Figure S5). The distribution of the average optimization accuracies of the randomized sets shows dramatically lower values than those of the original data, indicating that our optimization procedure, when based on a meaningful clinical stratification, is able to identify non-random and very specific gene-subgroup associations (Figure S6).

In the second analysis, we recursively performed the optimization procedure after excluding the identified minimal set of genes at each iteration. We observed a progressive decrease in accuracy and, as expected, higher values of optimal θ and λ in later iterations, indicating less effective gene selection and dimensionality reduction (Figure S7). Overall, we observed that this decay in accuracy upon iterative removal of selected genes can be divided into three phases: a short initial phase (accuracies between 94.94 and 88.57) in which large sets of genes are removed at each iteration (1027.72 on average), a long intermediate phase (accuracies between 79.76 and 69.96) in which less genes are removed (23.31 on average), and a short final phase (between 57.06 and 31.43) in which an average of 1.08 gene is removed at each iteration before the accuracy drops to 0. At the end of this procedure, the cumulative number of removed genes is 5,950.63 (average per patient; 38 patients). These results show the effectiveness of the greedy nature of our optimization algorithm, which is able to achieve high accuracies even when the pool of genes it operates upon is largely reduced.

### Sensitivity analyses

To test if our clusters are a good representation of the similarities among patients, we performed a sensitivity analysis with two approaches for clustering significance assessment. The first, based on multiscale bootstrap resampling (Suzuki and Shimodaira 2006), assigns a confidence value, known as approximately unbiased probability value (pvAU), to each cluster. High pvAU indicates high confidence in the clusters. The second, based on a Monte Carlo procedure (Kimes et al., 2017), assigns an empirical p value and a Gaussian approximate p value to each cluster. An important difference between the two approaches is that the multiscale bootstrap resampling approach tends to be less conservative than the Monte Carlo-based procedure, which outperformed the first with simulated and real-world data (Kimes et al., 2017).

At the root node, WNT and SHH subgroups are significantly separated from G3 and G4 subgroups with empirical p value of 1.08e-02 (Gaussian approximate p value of 2.59e-03) (Figures S8 and S9). Such two large partitions are poorly supported by the data (pvAU 49.23% and 63.05%, respectively), indicating the possibility of a finer subdivision. Indeed, WNT subgroup significantly separates from SSH subgroup with empirical p value of 5.45e-02 (Gaussian approximate p value of 3.55e-02), whereas G4 subgroup significantly separates from G3 subgroup with empirical p value of 1.85e-02 (Gaussian approximate p value of 6.75e-03).

Unlike the three main subgroups WNT (pvAU 100%), G4 (pvAU 99.97%), and G3 (pvAU 92.79%), SHH appears to be poorly supported by the data as a unique cluster (pvAU = 38.41%), whereas two SHH sub-clusters might exist (pvAU 99.88% and pvAU 99.55%, respectively), although their separation is not statistically significant (empirical p value 1.02e-01; Gaussian approximate p-value 9.74e-02). Of note, a finer partition of SHH subgroup into multiple sub-clusters has been reported by recent studies (Schwalbe et al., 2017; Archer et al., 2018).

The fifth cluster (G3-G4), despite being composed of two patients previously described as G4 (MB09 and MB54), and one as G3 (MB47), is supported by the data (pvAU 83.98%), but its separation from the G3 subgroup is not statistically significant (empirical p value 1.01e-01; Gaussian approximate p value 9.18e-02). Interestingly, patients of this cluster were all assigned to G4 via network fusion and to G3 only using methylation data (Forget et al., 2018). Indeed, an overlap of genetic features between G3 and G4 has also been reported by a study on risk stratification (Schwalbe et al., 2017).

Overall, these sensitivity analyses indicate that (1) 4 of 5 clusters found in our optimization procedure are statistically significant based on a Monte Carlo approach (Kimes et al., 2017) and recapitulate the classical MB molecular subgroups and (2) the small fifth cluster (G3-G4) shares similarities with G3 whose heterogeneity was previously observed (Schwalbe et al., 2017; Forget et al., 2018).

### Provenance analysis of the identified gene communities

By performing a network enrichment analysis test (Signorelli et al. 2016), we identified the most significantly overrepresented intra-layer edges among the genes of the minimal sets identified for each patient in each cluster (see methods: “multilayer network enrichment analysis”). In the following, we analyze those associations that are unique of the five clusters and enriched in all patients of each cluster (Table S6). Beside this strict requirement, several other enriched associations are shared among clusters and can be further explored (see resource availability: “data and code availability”). Overall, we found that the minimal set of genes found in all patients of WNT, SHH, and G4 clusters are uniquely enriched in very specific associations in each layer, whereas G3-G4 and G3 clusters tend to display less specific enrichments (i.e., either several or no enriched associations). This reduction of enrichment specificity from WNT to G3 suggests an interesting parallel with the prognosis spectrum of the four classical subgroups, from best (WNT) to worst (G3) outcomes.

• **Molecular associations.** As for the molecular interaction layer, WNT cluster presents enrichment in four proteins: ACVR2A, a receptor involved in the activin signaling pathway (Chen et al., 2006), which is also enriched in this cluster in the pathways layer; ATP4A, a subunit of the ATPase H^+^/K^+^, a membrane transporter that is target of the Hedgehog signaling pathway, whose low levels of β1 subunit have been related to cell proliferation in MB models (Lee et al., 2015); POU2F2, which has been recently found to play a role in spinal cord development in a mouse model (Masgutova et al., 2019) and suspected to be regulated by miRNAs in MB (Venkataraman et al., 2013); and RBM48, a protein found to be amplified across several cancer tissues and cell lines and that may have a role in apoptotic processes (Hart et al., 2015). SHH cluster is uniquely enriched in molecular interactions of various gene products, including two proto-oncogenes (*ETS1* [Cao et al., 2015] and *JUND* [Elliott et al., 2019]), a calcium voltage-gated channel (CACNA1A) significantly downregulated in MB and other brain tumors (Phan et al., 2017), and interestingly a long noncoding RNA (*LINC00461*), expressed predominantly in the brain and involved in tumorigenesis (Yang et al., 2017). G4 cluster only presents enrichment in the interactions of ARID4A, a member of the ARID family such as ARID1B, a repressor of Wnt/β-catenin signaling (Vasileiou et al., 2015). G3 cluster is enriched in interactions of the ABC transporter, ABCA3, suspected to be involved in chemoresistance in brain tumor progression (Hadjipanayis and Van Meir 2009); the dystrophin-glycoprotein SGCB; the SUMO ligase PIAS1, which increases the activity of Gli proteins on the Hedgehog pathway (Niewiadomski et al., 2019); and the heat shock protein DNAJB5, which regulates histone deacetylase (HDAC) nuclear shuttling, whose inhibition is considered to be a promising therapy in MB (Becher 2019).
• **Drug-target associations.** As for the drug layer, G3 cluster is the only one showing a unique enrichment in all patients, namely, in lubeluzole, an inhibitor of nitric oxide (NO) synthesis (Maiese et al. 1997). This observation points toward the role of oxidative stress in MB under the light of results from NO synthesis inhibition in experimental models (Haag et al., 2012) and clinical trials in G3 subgroup (Bakhshinyan et al., 2019).
• **Variant-disease associations.** As for the disease layer, the enriched associations may indicate overlapping features between MB and molecular processes underlying other pathologies. WNT cluster is uniquely associated with alveolar rhabdomyosarcoma, a common soft tissue sarcoma in children (Barr 2011), and familial prostate carcinoma. The implication of the overactivation of the Hedgehog signaling pathway in both MB and rhabdomyosarcoma (Azatyan et al., 2019) as well as in prostate cancer (Amakye et al. 2013; Ng and Curran 2011) has been extensively reported. SHH cluster is uniquely associated with macular degeneration and syndromic craniosynostosis, also characterized by ocular abnormalities, suggesting a link with the ophthalmic complications of MB, which occur as a result of the disease and its treatments (Cassidy et al., 2000). Polydactyly (Crane et al., 2018) appears to be uniquely enriched in G4 cluster, MB being a feature of several disorders of infants often characterized by akin skeletal abnormality such as Gorlin syndrome (Lo Muzio 2008) and others (Osterling et al., 2011). Cluster G3-G4 shows many enriched diseases, including several cancers, forms of hypogonadism, and interestingly Dravet syndrome, a genetic disorder that causes severe epilepsy in infants. Of note, MB is among the most frequent tumors of cerebellum presenting with seizures (5%) (Sánchez Fernández and Loddenkemper, 2017). G3 does not display unique enrichments in the disease layer.
• **Pathway associations.** As for the pathway layer, the WNT cluster is uniquely enriched in cell differentiation in early embryogenesis, such as Nodal (Brown et al., 2011) and Activin (Chen et al., 2006) signaling; immune response, such as Dendritic cell-associated C-type lectin-2 (Dectin-2) carbohydrates receptor activity (Graham and Brown 2009); protein metabolism, such as insulin-like growth factor (IGF) regulation (Holly and Perks 2006); and defects in the mismatch repair (MMR) system (Chao and Lipkin 2006). SHH cluster is uniquely enriched in potassium channels of the neuronal system, such as the Kir channel (Radeke et al. 1999), and signal transduction, such as calcitonin (Sexton et al. 1999) and Hedgehog (Briscoe and Thérond 2013) signaling. G4 is associated with fusion events in the *FGFR1* gene (Braun and Shannon 2004) and neuronal system transmission, such as excitatory synaptic transmission by glutamate receptors (Kessels and Malinow 2009). The great majority of these pathways have been directly or indirectly related to MB in the literature, such as the interplay between the embryonic morphogens Nodal and Hedgehog in brain development (Rohr et al., 2001), the activation of Activin signaling in a subset of G3 subgroup (Morabito et al., 2019), the role of *FGFR1* in gliomas (Egbivwie et al., 2019), and the importance of carbohydrate antigen recognition in MB (Read et al., 2009). Clusters G3-G4 and G3 show a varied landscape of enriched pathways.
• **Metabolic reaction associations.** As for the metabolome layer, uniquely enriched metabolites of the WNT cluster are ferricytochrome C (part of mitochondrial respiratory electron transport chain), nicotinamide nucleotide (a derivative of niacin, a form of vitamin B3), superoxide anion (a reactive oxygen species), and ribose 5-phosphate (a precursor to many biomolecules, including DNA and RNA). SHH shows unique enrichments for nicotinate D-ribonucleotide (part of cofactor biosynthesis) and pantothenate (vitamin B5), whereas G4 is uniquely enriched in sulfate (the major sulfur source in humans). Cluster G3 does not present uniquely enriched metabolites, whereas G3-G4 shows several.

### Method verification on an independent cohort

To further verify the applicability of our methodology, we performed the same analytical procedure on an independent, non-overlapping, multi-omics MB cohort (Archer et al., 2018) (see methods: “data sources of medulloblastoma genes”). This cohort study collects proteogenomics data from 45 patients and proposes a finer categorization of SHH and G3 subgroups (SHHa, SHHb, G3a, G3b). A total of 39 patients display complete multi-omics information, whereas 6 lack RNA sequencing, including all 3 patients of the WNT subgroup.

In a first analysis, we were able to recapitulate the 5 clusters (SHHa, SHHb, G3a, G3b, G4) of the 39 patients with complete multi-omics information, achieving the highest accuracy (98.29%, MCC = 0.95) with optimized parameters λ = 3 and θ = 0 (Figure S10), which corresponds to an average of 842.2 genes per patient (SD = 145.12) and average dimensionality reduction of 92.83% (SD = 0.578). All patients are correctly assigned to their subgroups, whereas only MB136, labeled as a SHHb member, clusters with the SHHa subgroup.

As for the previous analysis, the patients with incomplete multi-omics information were used as validation set and assigned individually to subgroups based on the Jaccard Index (J) (see methods: “identification of the minimal set of genes that define medulloblastoma subgroups”). Patients MB037, MB018, and MB282 are correctly classified as SHHa, G3a, and G4, the most similar patients being MB239 (J = 0.177), MB226 (J = 0.136), and MB091 (J = 0.166), respectively.

In a second analysis, we included all 45 patients achieving the highest accuracy (95.56%, MCC = 0.85) with 7 clusters and λ = 5 and θ = 1 (Figure S11), which corresponds to an average of 1,073.58 genes per patient (SD = 161.94) and average dimensionality reduction of 90.59% (SD = 1.06). The performance reduction suggests that the addition of patients presenting missing data in the parameters optimization procedure can decrease its performance.

## Discussion

Molecular disease subtyping is a fundamental tool to achieve an effective patient stratification for clinical trials and preventive and therapeutic interventions. In some cancers, such as breast cancer and blood cancers, subtyping has been very successful thanks to the statistical power brought by cohorts composed of large numbers of patients. Rare diseases represent a more challenging setting because, by definition, they affect a small number of patients with studies that, in most cases, are in the order of tens of subjects. MB, such as other pediatric cancers, is an illustrative example, two MB subgroups being very well distinguishable (SHH and WNT) and two others being far less characterized (G3 and G4).

In our vision, a meaningful molecular subtyping of rare diseases can be achieved by leveraging the wealth of biomedical information that is available in public knowledge bases and that can be integrated in the form of multilayer networks. In particular, achieving patient stratification by means of structural features (multilayer community trajectories) extracted from a general-purpose multilayer network represents a way to both identify the minimal set of genes that characterize the subgroups and, most importantly, to obtain information about the types of relations that define the associations of such genes (e.g., targeting drugs, pathways, molecular interactions). This way of accomplishing two objectives with one action constitutes the main achievement of our methodology.

In this regard, this work is additionally motivated by the relevance and urgency of implementing computational solutions based on biological multilayer networks. Borrowing from social network science, we use multiplexity as a way to evaluate intimacy of gene associations in MB: the more tightly a group of genes is connected through multiple types of features, the more clearly defined and explainable that community will be (Dickison et al. 2016).

Our results show that we can accurately recapitulate the four established MB subgroups using proteogenomic data and correctly classify the patients with partial molecular profiles. The approach enables an effective dimensionality reduction leading to the identification of a minimal set of altered genes that are sufficient to define MB subgroups. Moreover, the use of a multilayer network in this context allows the retrieval and analysis of the multiple associations among the identified genes, enabling a high level of interpretation of the patient subgroups and the spectrum of prognosis that characterize them, from best (WNT) to worst (G3) outcomes. Analyzing the provenance of the associations that determine the detected communities is extremely beneficial to better characterize the molecular determinants of the patient subgroups and, in turn, achieve a high level of explainability, a matter of considerable debate in computational biology lately (Adadi and Berrada 2020).

An additional important aspect that emerges from our results is that the precise clinical stratification of patients and the completeness of multi-omics information can lead to a better optimization and finer molecular characterization. Indeed, the overall performances of our optimization approach, in terms of both clustering accuracy and dimensionality reduction, are higher using a patient stratification of reference of six subgroups (Archer et al., 2018) compared with the traditional four subgroups (Forget et al., 2018). This indicates that precise clinical hypotheses can lead to precise molecular characterization of patient subgroups, making multilayer networks a powerful and unique tool especially for the study of rare diseases.

### Limitations of the study

The main limitations of the study include (1) the scope of the multilayer network, (2) the reliability of the patient stratification of reference, and (3) the suitability of modularity as a quality function for community detection. As for the multilayer network, we distilled high-quality information from reputable and widely used knowledge bases (see methods: “data sources for the construction of the multilayer network”). Our multilayer network encapsulates a comprehensive view of fundamental aspects of human biology, but it can be further expanded to layers with a different content. As for the patient stratification of reference, the categorization of the cohort under study, based on network fusion (Forget et al., 2018), is one of the most recent and highly accurate attempts to cluster patients with MB using multi-omics information. Our analysis can be repurposed for different MB cohorts, available at data sharing platforms such as R2 (http://r2.amc.nl) and Cavatica (www.cavatica.org), among others. As for modularity, it is one of the most well-known quality functions for community detection (Chen et al., 2018). Moreover, the Louvain algorithm has been adapted for multilayer networks (Didier et al. 2018; Didier et al. 2015). Nevertheless, our approach can be applied to other quality functions (e.g., Hamiltonians, partition density) and more recent algorithms, such as the Leiden algorithm (Traag et al. 2019), which, to our knowledge, has currently not been adapted to multilayer networks.

### Resource availability

#### Lead contact

Further information and requests for resources and reagents should be directed to and will be fulfilled by the lead contact, Davide Cirillo (davide.cirillo@bsc.es).

#### Materials availability

This study did not generate reagents, cell lines, or any biological material.

#### Data and code availability

The data and code generated during this study is available at dedicated GitHub repositories. The developed CmmD package is available at https://github.com/ikernunezca/CmmD. The code to reproduce all the figures and tables is available at https://github.com/ikernunezca/Medulloblastoma, where the complete lists of network enrichments and the processing of MB gene lists from the cohorts under study are also available. The text mining process is automated in the workflow available at https://github.com/cirillodavide/ipc_textmining. The procedure to generate the multilayer network used in this work is available at https://github.com/cirillodavide/gene_multilayer_network.

## Methods

All methods can be found in the accompanying transparent methods supplemental file.

## Supplemental information

Document S1. Transparent methods, figures S1–S11, and tables S1–S4

Table S5. Minimal set of genes, related to figure 5 Minimal sets of altered genes associated with each one of the 38 patients with medulloblastoma from Forget et al. (2018). The labels of the original subgroups (clusters) and the ones assigned after the optimization procedure are reported.

Table S6. Multilayer network enrichment analysis, related to figure 5 The table reports those associations (edges) among the minimal sets of genes that are enriched in all the patients of a cluster and unique of each cluster (WNT, SHH, G3, G4, G3-G4) for a specific layer of the multilayer network (see methods: “multilayer network enrichment analysis”). Association IDs are grounded in databases (see methods: “data sources for the construction of the multilayer network”).
