# Supplementary material for: The multilayer community structure of medulloblastoma
Source: iScience. 2021 Mar 26;24(4):102365. doi: 10.1016/j.isci.2021.102365 (PMC8050854; doi:10.1016/j.isci.2021.102365)
Supplement: Document S1. Transparent methods, figures S1–S11, and tables S1–S4 [file mmc1.pdf]

iScience, Volume *24*

Supplemental information

The multilayer community structure of medulloblastoma

Iker Núñez-Carpintero, Marianyela Petrizzelli, Andrei Zinovyev, Davide Cirillo, and Alfonso Valencia

Supplemental Figures

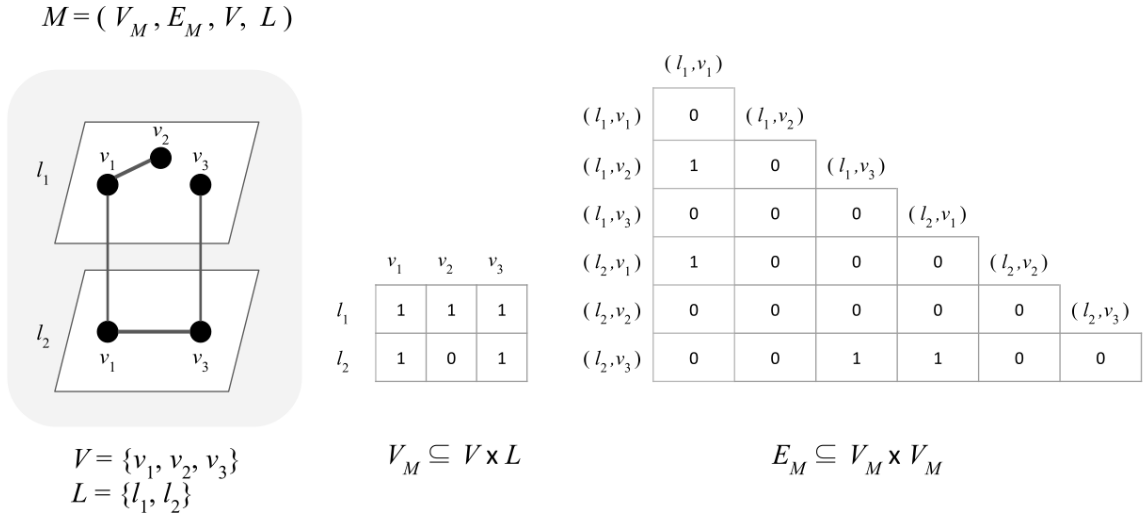

Figure S1. Multilayer network definition, Related to Figure 1 and Figure 2. A multilayer network M, such as the one represented inside the grey area, is defined as a quadruplet of four elements (V_M, E_M, V, and L). V and L are the sets of nodes and layers of M, respectively. V_M and E_M are the sets of nodes contained in each layer and edges connecting them within (intra-layer) and between (inter-layer) layers, respectively. As the one represented here, we build a multilayer network where inter-layer edges only connect the same nodes in each layer.

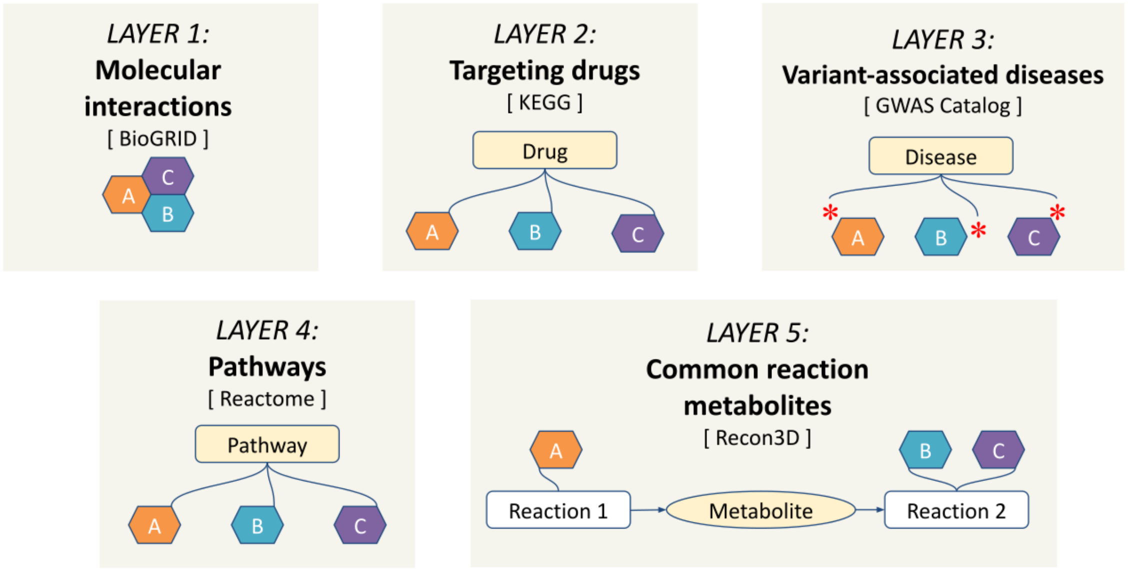

Figure S2. Gene-gene association represented in the fiver layers of the multilayer network, Related to Figure 1 and Figure 2. Gene entities are represented as hexagons. Associations retrieved from the databases in squared parentheses are represented as curved lines. Red asterisks indicate mutations.

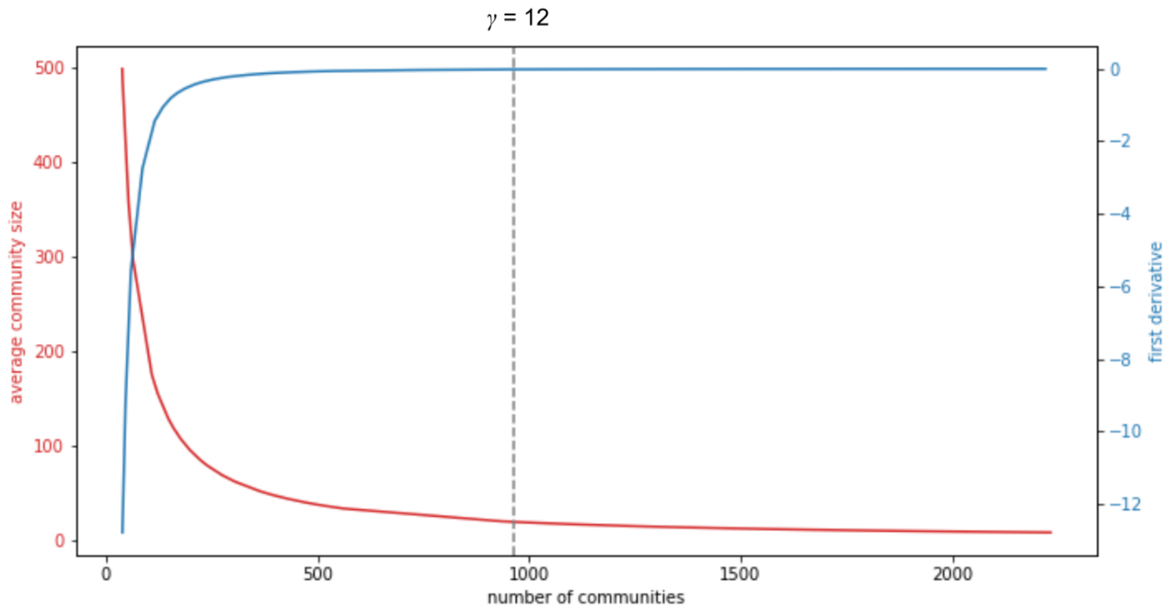

Figure S3. Identification of the resolution range of interest, Related to Figure 2. The modularity resolution parameter (γ) determines the number of communities and their size. The most dramatic changes in both size and number of communities occur in an initial range of resolution, which enables to detect genes that are strongly associated. We identified the endpoint of this range (γ = 12) as the value where the average community size, as a function of the number of communities, establishes a plateau (i.e. its first derivative equals zero with 0.05 margin of error).

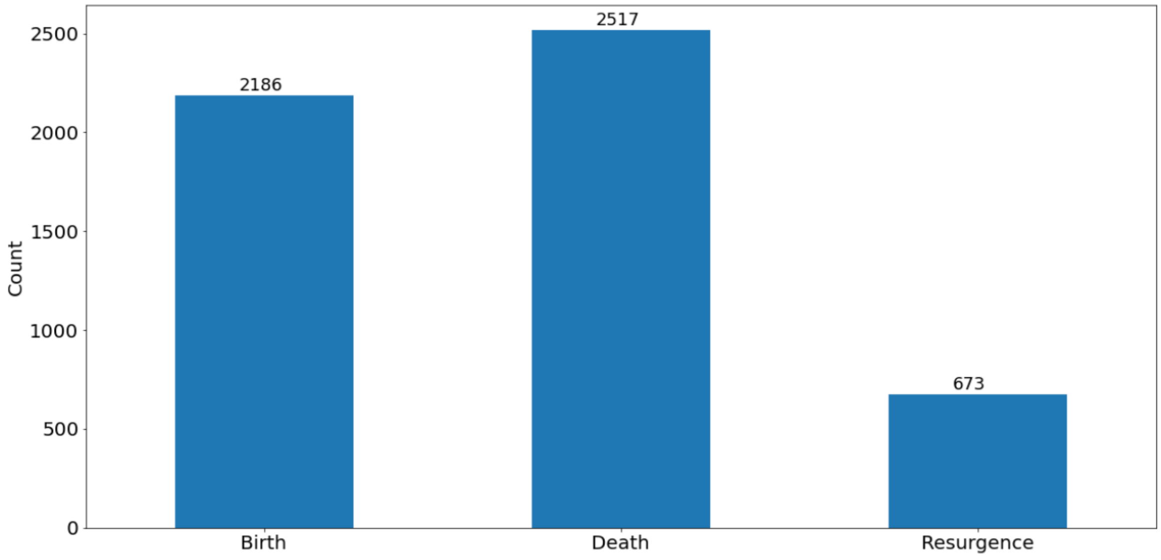

Figure S4. Operations on dynamic communities, Related to Figure 3. Count of dynamic events (birth, death, and resurgence) in the multilayer communities that contain text-mined medulloblastoma genes.

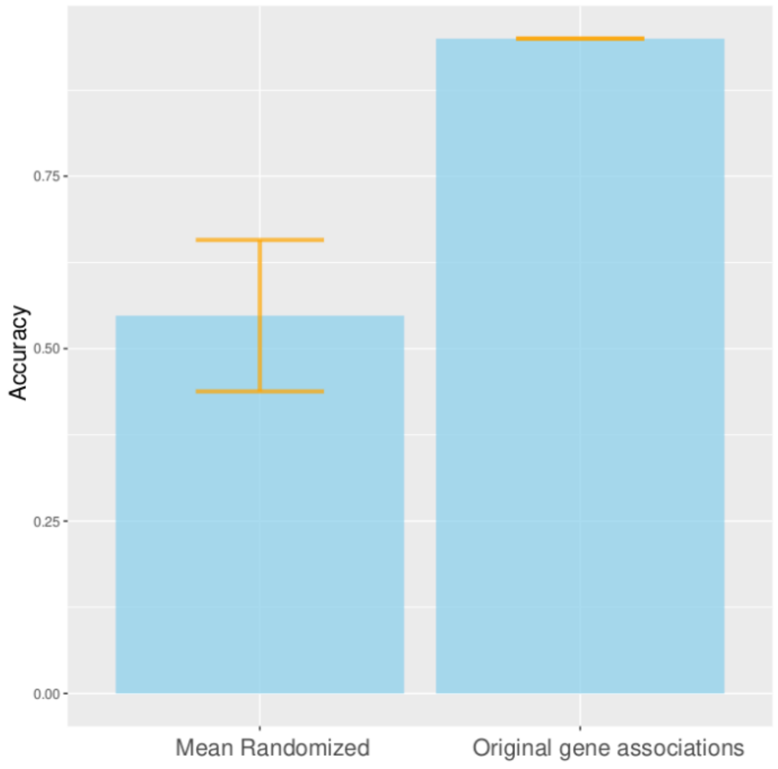

Figure S5. Gene shuffling test, Related to Figure 4 and Figure S6. The bar plots show the comparison between the highest accuracy achieved with the optimization procedure (94.94%, “Original gene associations”) and the average accuracy achieved by shuffling the genes in the cohort 10,000 times (54.76%, SD = 0.11, “Mean Randomized”), maintaining the same number of genes for each patient as in the original data and using the optimal parameters $\theta = 0$ and $\lambda = 6$.

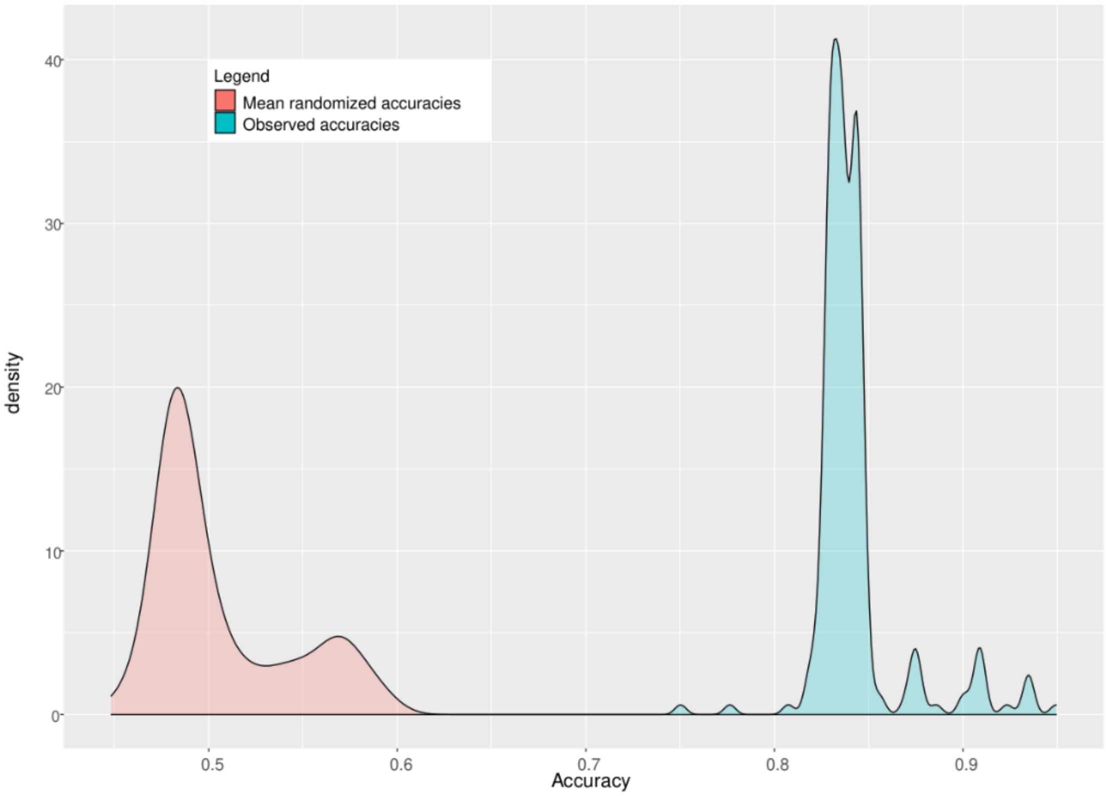

Figure S6. Distributions of optimization accuracies, Related to Figure 4 and Figure S4. The distribution of the optimization accuracies in the original data is reported in green, and the distribution of the average optimization accuracies after shuffling the altered genes across the cohort 10,000 times, maintaining the same number of genes for each patient is reported in red.

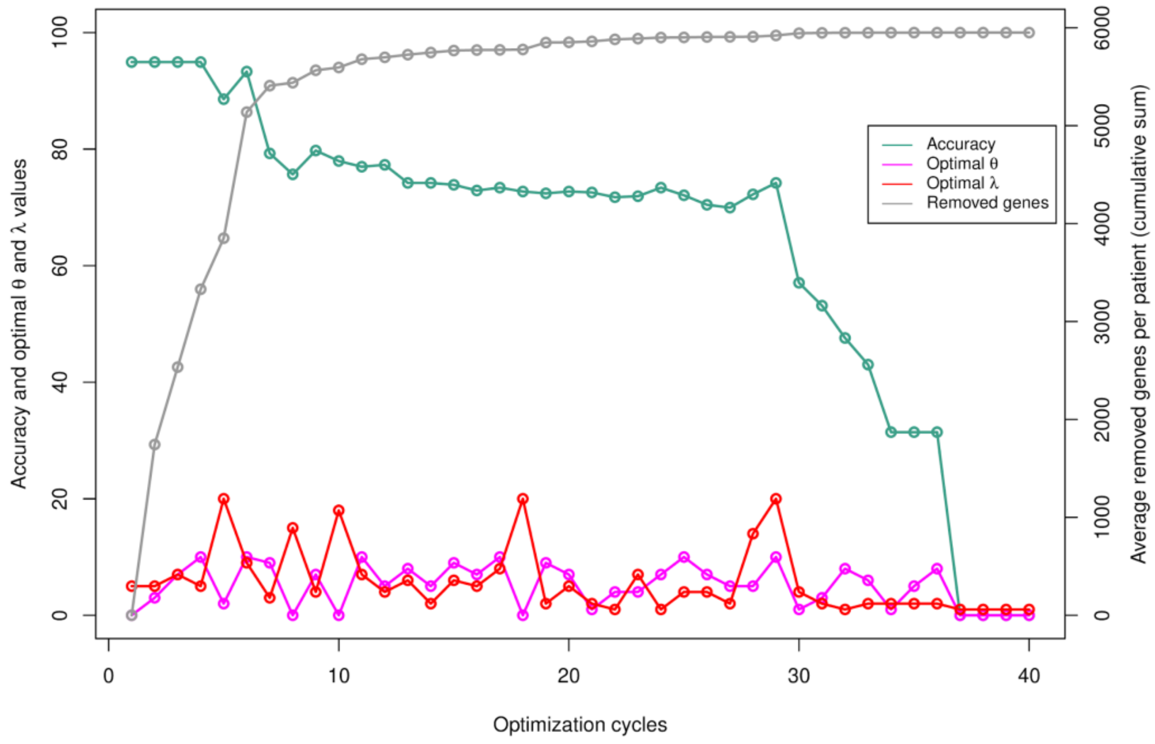

Figure S7. Recursive exclusion test, Related to Figure 4. The plot shows the iterative removal of selected genes in the cohort of 38 medulloblastoma patients. At every iteration, the minimal set of genes, found at optimal values of θ (purple line) and λ (red line) corresponding to highest accuracy (green line), is removed and the optimization procedure is repeated. The cumulative average number of genes per patient that are removed at every iteration is reported (grey line).

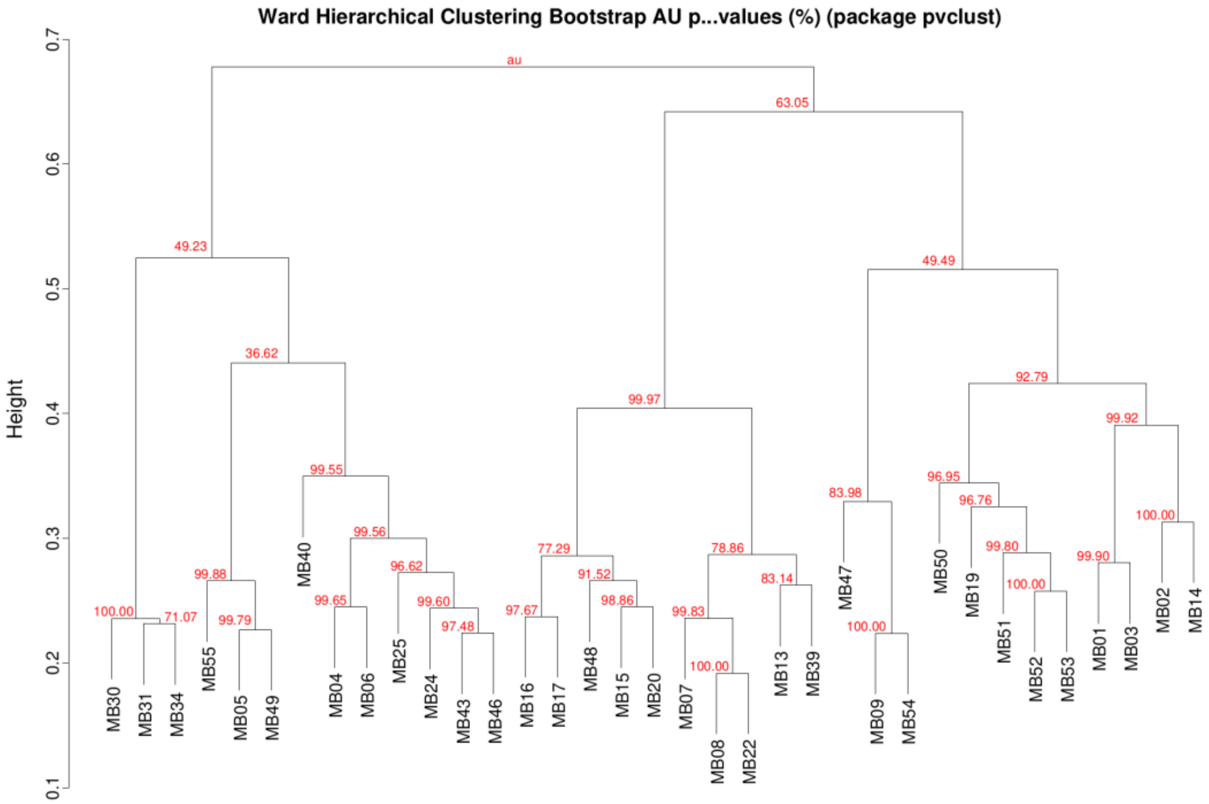

Figure S8. Clustering significance, Related to Figure 5. Significance assessment of hierarchical clustering (Ward method) of medulloblastoma patients using multiscale bootstrap resampling (Suzuki and Shimodaira, 2006). AU p values (%), or approximately unbiased probability value (pvAU), is reported in red on top of each cluster.

A

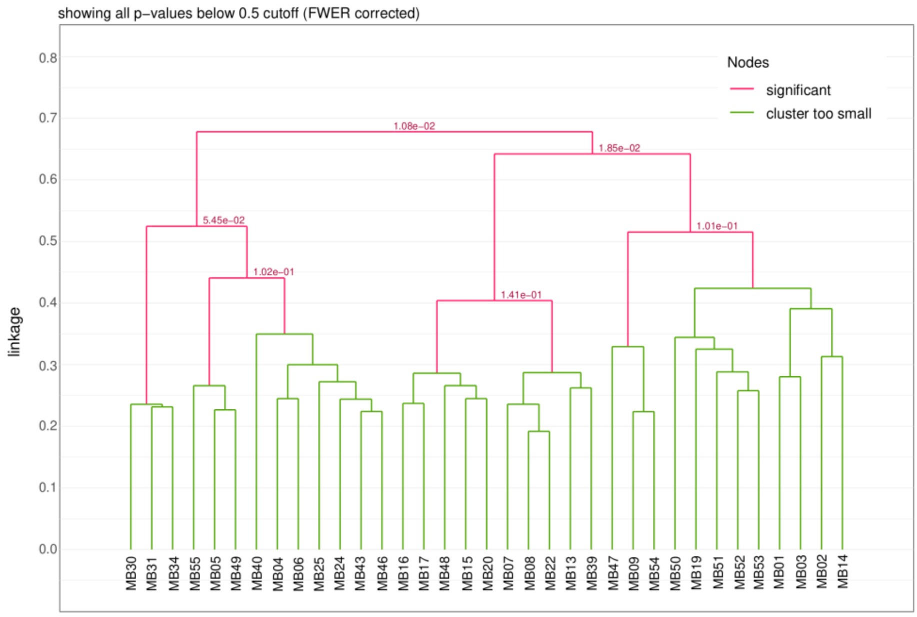

B

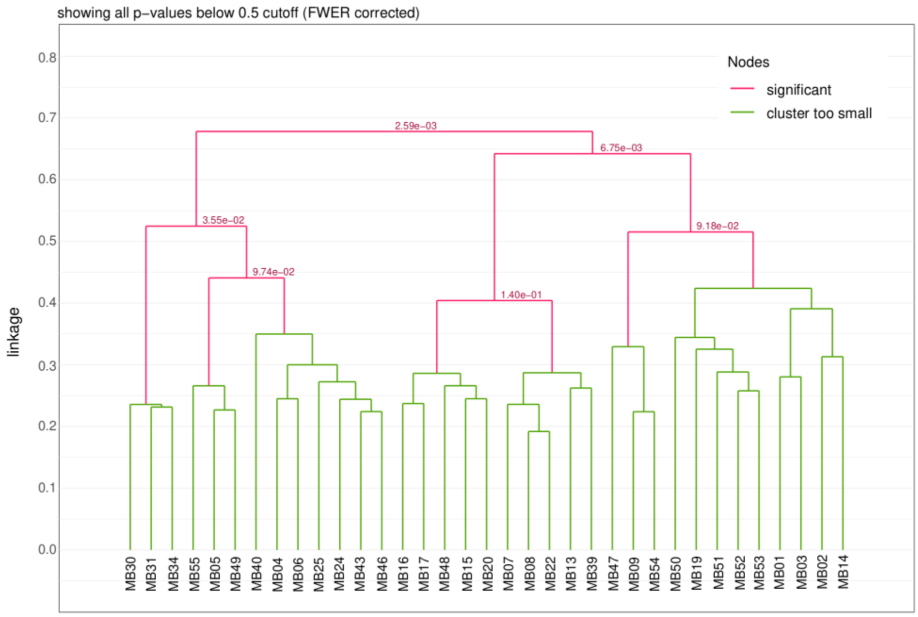

Figure S9. Clustering significance, Related to Figure 5. Significant assessment of hierarchical clustering (Ward method) of medulloblastoma patients using a Monte Carlo procedure (Kimes *et al.*, 2017). (A) empirical p-value and (B) Gaussian approximate p-value are reported in red on top of each cluster.

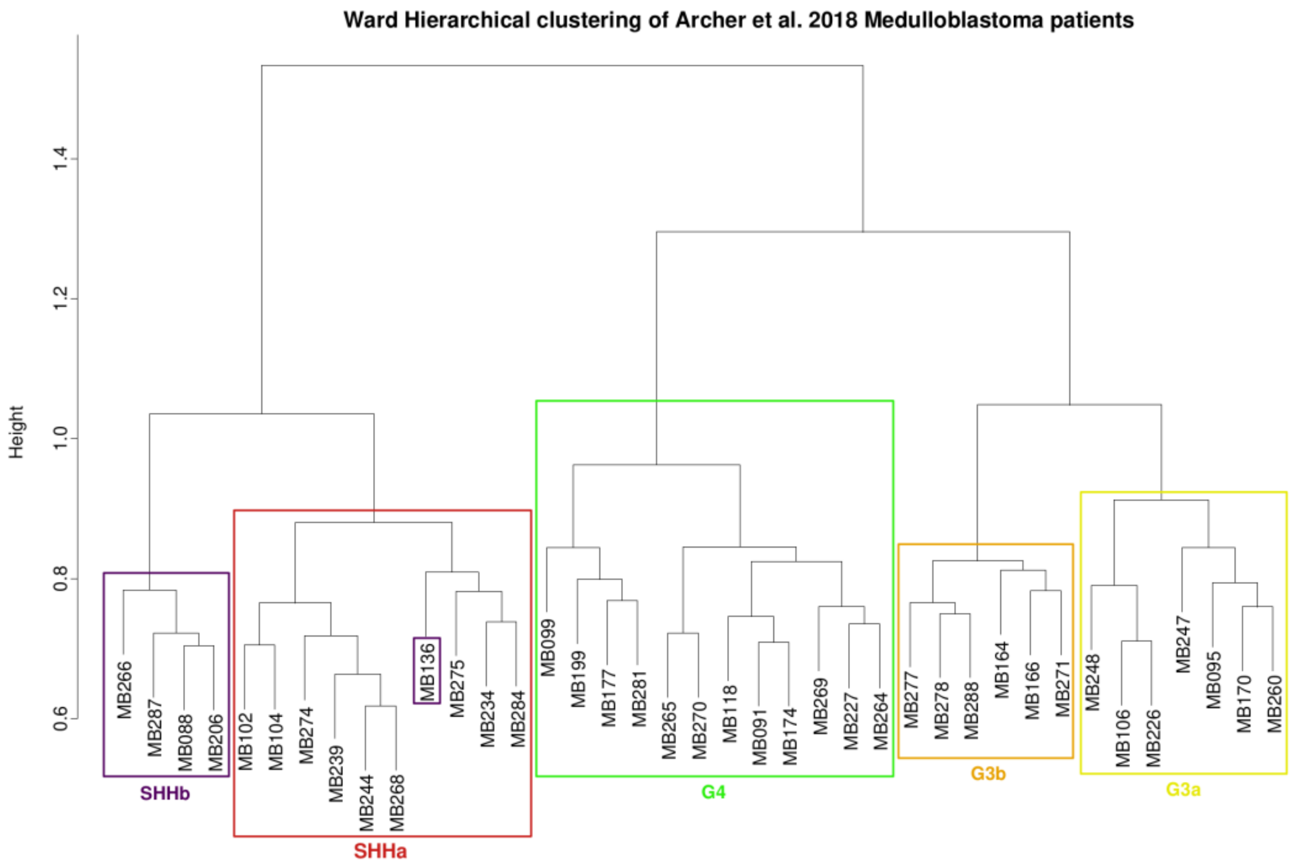

Figure S10. Hierarchical clustering of medulloblastoma patients from Archer et al. 2018, Related to Figure 2 and Figure S11. Ward's linkage hierarchical clustering obtained at $\lambda = 3$ and $\theta = 0$ for patients with complete multi-omics data (Archer *et al.*, 2018). Rectangles indicate the 5 clusters suggested by PAM (partitioning around medoids) criteria. The color of each cluster indicates the original patient stratification into the five medulloblastoma subgroups: SHHa (red), SHHb (purple), Group 4 (G4, green), Group 3a (G3, yellow), Group 3b (G3b, orange). Patient MB136, originally labeled as SHHb subgroup and highlighted with a purple lower level rectangle, clusters within the SHHa subgroup.

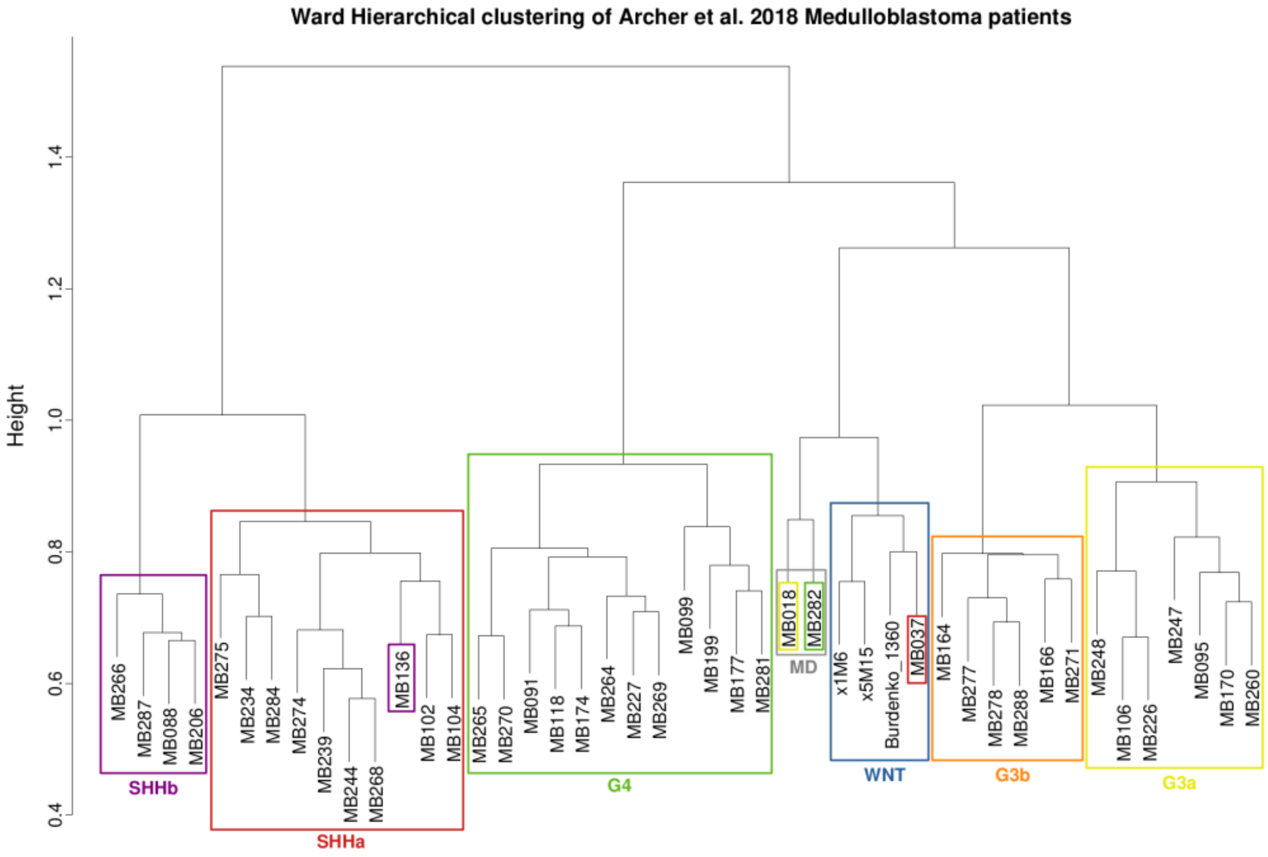

Figure S11. Hierarchical clustering of medulloblastoma patients from Archer et al. 2018, Related to Figure 2 and Figure S10. Ward's linkage hierarchical clustering obtained at $\lambda = 5$ and $\theta = 1$ for patients with complete and incomplete multi-omics data (Archer *et al.*, 2018). Rectangles indicate the 7 clusters suggested by PAM (partitioning around medoids) criteria. The color of each cluster indicates the original patient stratification into the six medulloblastoma subgroups: WNT (blue), SHHa (red), SHHb (purple), Group 4 (G4, green), Group 3a (G3, yellow), Group 3b (G3b, orange). Patients with missing data cluster together (MD, Missing Data). Misclassified patients are highlighted with lower level rectangles indicating their original subgroup.

Supplemental Tables

Table S1. Optimal number of clusters, Related to Figure 4. The matrix shows the optimal number of clusters, based on the partitioning around medoids (PAM) algorithm, for combinations of parameters θ (rows) and λ (columns).

λ

θ	1	2	3	4	5	6	7	8	9	10	11	12	13	14	15	16	17	18	19	20
0	9	10	7	9	9	5	10	6	8	8	10	8	10	9	8	8	9	8	9	6
1	8	10	10	10	9	8	10	8	8	9	8	8	8	8	8	8	9	9	10	8
2	8	8	8	10	8	9	8	10	9	10	8	10	9	9	8	8	8	9	8	10
3	10	10	9	5	9	9	5	10	10	9	10	8	8	10	8	10	10	8	8	8
4	10	10	10	4	8	9	5	10	10	5	9	9	5	5	5	10	8	8	8	8
5	10	8	9	7	8	8	5	10	10	10	9	5	8	6	10	10	8	9	8	8
6	8	9	10	7	9	8	8	10	10	5	9	4	9	6	10	9	9	9	8	8
7	7	9	7	7	7	7	9	8	10	8	9	5	10	10	5	7	9	5	8	8
8	8	8	8	8	8	8	9	4	8	8	8	8	10	10	10	10	9	9	8	8
9	8	7	8	8	8	8	9	4	8	8	4	5	8	10	4	4	9	9	10	10
10	8	9	8	8	9	9	9	8	8	9	10	10	9	9	9	10	9	5	8	10

Table S2. Optimization accuracies, Related to Figure 4. The matrix shows the accuracies of the optimization procedure (see Methods: “Identification of the minimal set of genes that define medulloblastoma subgroups”) for combinations of parameters θ (rows) and λ (columns). The maximum accuracy achieved is highlighted in bold.

λ

θ	1	2	3	4	5	6	7	8	9	10	11	12	13	14	15	16	17	18	19	20
0	0.842	0.819	0.886	0.829	0.837	**0.949**	0.819	0.874	0.873	0.835	0.824	0.835	0.827	0.833	0.843	0.843	0.837	0.829	0.837	0.9
1	0.84	0.827	0.83	0.827	0.837	0.843	0.83	0.843	0.843	0.837	0.837	0.843	0.847	0.847	0.843	0.843	0.848	0.837	0.83	0.843
2	0.835	0.843	0.847	0.835	0.843	0.837	0.847	0.83	0.83	0.83	0.843	0.83	0.833	0.837	0.843	0.843	0.847	0.833	0.843	0.827
3	0.835	0.83	0.829	0.832	0.829	0.837	0.909	0.83	0.824	0.837	0.83	0.843	0.843	0.83	0.847	0.83	0.83	0.843	0.843	0.843
4	0.835	0.83	0.83	0.869	0.843	0.829	0.909	0.83	0.83	0.909	0.833	0.837	0.9	0.909	0.91	0.83	0.835	0.835	0.843	0.843
5	0.832	0.843	0.829	0.835	0.835	0.843	0.75	0.819	0.83	0.83	0.829	0.909	0.835	0.874	0.819	0.827	0.847	0.837	0.843	0.843
6	0.847	0.833	0.824	0.837	0.837	0.843	0.843	0.83	0.83	0.835	0.837	0.935	0.837	0.874	0.835	0.829	0.833	0.829	0.837	0.843
7	0.876	0.832	0.835	0.876	0.835	0.835	0.833	0.833	0.83	0.843	0.833	0.909	0.83	0.83	0.835	0.835	0.837	0.776	0.829	0.843
8	0.838	0.845	0.847	0.843	0.842	0.847	0.837	0.856	0.847	0.843	0.843	0.843	0.83	0.83	0.83	0.84	0.827	0.838	0.847	0.843
9	0.842	0.876	0.847	0.835	0.843	0.843	0.837	0.807	0.843	0.843	0.935	0.835	0.843	0.83	0.935	0.935	0.837	0.843	0.829	0.83
10	0.837	0.832	0.847	0.847	0.829	0.837	0.829	0.843	0.842	0.829	0.83	0.83	0.837	0.835	0.829	0.824	0.829	0.923	0.855	0.824

Table S3. Optimization MCC, Related to Figure 4. The matrix shows the Matthews Correlation Coefficient (MCC) of the optimization procedure (see Methods: “Identification of the minimal set of genes that define medulloblastoma subgroups”) for combinations of parameters θ (rows) and λ (columns). The maximum MCC value achieved is highlighted in bold.

λ

θ	1	2	3	4	5	6	7	8	9	10	11	12	13	14	15	16	17	18	19	20
0	0.603	0.536	0.719	0.564	0.590	**0.876**	0.536	0.682	0.685	0.582	0.554	0.582	0.563	0.581	0.608	0.608	0.590	0.564	0.590	0.754
1	0.596	0.563	0.572	0.563	0.590	0.608	0.572	0.608	0.608	0.590	0.590	0.608	0.617	0.617	0.608	0.608	0.624	0.590	0.572	0.608
2	0.582	0.608	0.617	0.586	0.608	0.590	0.617	0.572	0.572	0.572	0.608	0.572	0.578	0.590	0.608	0.608	0.617	0.581	0.608	0.563
3	0.589	0.572	0.564	0.577	0.564	0.590	0.771	0.572	0.554	0.590	0.572	0.608	0.608	0.572	0.617	0.572	0.572	0.608	0.608	0.608
4	0.589	0.572	0.572	0.678	0.608	0.564	0.771	0.572	0.572	0.771	0.578	0.590	0.751	0.771	0.776	0.572	0.582	0.582	0.608	0.608
5	0.577	0.608	0.564	0.575	0.582	0.608	0.382	0.536	0.572	0.572	0.564	0.771	0.582	0.682	0.536	0.563	0.617	0.590	0.608	0.608
6	0.617	0.581	0.554	0.579	0.590	0.608	0.608	0.572	0.572	0.582	0.590	0.841	0.590	0.682	0.589	0.564	0.581	0.564	0.590	0.608
7	0.694	0.577	0.575	0.694	0.575	0.575	0.581	0.578	0.572	0.608	0.581	0.771	0.572	0.576	0.582	0.575	0.590	0.421	0.564	0.608
8	0.595	0.612	0.617	0.608	0.603	0.617	0.590	0.643	0.617	0.608	0.608	0.608	0.572	0.572	0.572	0.603	0.563	0.595	0.617	0.608
9	0.603	0.694	0.617	0.582	0.608	0.608	0.590	0.521	0.608	0.608	0.841	0.582	0.608	0.572	0.841	0.841	0.590	0.611	0.567	0.572
10	0.587	0.577	0.617	0.617	0.564	0.590	0.564	0.608	0.603	0.564	0.572	0.572	0.590	0.586	0.564	0.554	0.564	0.810	0.642	0.554

Table S4. Classification of patients with partial datasets, Related to Figure 5. The table reports the values of the Jaccard Index (J), parametrized by the optimal θ and λ, between the 3 patients with partial datasets (MB10, MB21, MB33) and the 35 patients with complete datasets (see Methods: “Data sources of medulloblastoma genes”).

	"MB10"	"MB21"	"MB33"
"MB01"	0.20855106888361	0.219810040705563	0.199903194578896
"MB02"	0.226933830382106	0.232198142414861	0.202247191011236
"MB03"	0.230385487528345	0.246086956521739	0.203089504770559
"MB04"	0.236396890717878	0.247598253275109	0.193577566711895
"MB05"	0.224057602710716	0.236489232019504	0.185480486781368
"MB06"	0.2255299954894	0.239740820734341	0.190839694656489
"MB07"	0.247404063205418	0.241379310344828	0.191169977924945
"MB08"	0.255896751223854	0.245812395309883	0.191443388072602
"MB09"	0.234858387799564	0.238391376451078	0.19559585492228
"MB10"	1	0.387596899224806	0.417813765182186
"MB13"	0.252108716026242	0.240667545015371	0.196420376319413
"MB14"	0.224178962398858	0.229138475417231	0.200670498084291
"MB15"	0.245346062052506	0.238565022421525	0.199434229137199
"MB16"	0.260057471264368	0.244523915958873	0.208097928436912
"MB17"	0.256641366223909	0.238726790450928	0.205223880597015
"MB19"	0.226726057906459	0.23021582733813	0.196706720071206
"MB20"	0.245344506517691	0.237636761487965	0.19560238204306
"MB21"	0.387596899224806	1	0.47027027027027
"MB22"	0.263229308005427	0.251486830926083	0.197016235190873
"MB24"	0.228245363766049	0.235772357723577	0.198476915754403
"MB25"	0.219874100719424	0.225834046193328	0.190647482014389
"MB30"	0.225081890500702	0.260118235561619	0.216873212583413
"MB31"	0.219325842696629	0.265342163355408	0.208029197080292
"MB33"	0.417813765182186	0.47027027027027	1
"MB34"	0.231185218566922	0.263134851138354	0.210621879255561
"MB39"	0.243792325056433	0.246463780540077	0.193433895297249
"MB40"	0.210699202252464	0.221179624664879	0.191943127962085
"MB43"	0.214088397790055	0.220472440944882	0.188539741219963
"MB46"	0.232285312060066	0.232372505543237	0.200093720712277
"MB47"	0.208278291501541	0.229626485568761	0.183826778612461
"MB48"	0.236533957845433	0.244097995545657	0.194895591647332
"MB49"	0.216193656093489	0.234702093397746	0.172910662824208
"MB50"	0.208942390369733	0.227743271221532	0.176949443016281
"MB51"	0.22202565236621	0.242437153813379	0.195921985815603
"MB52"	0.232150678931231	0.253062948880439	0.194782608695652
"MB53"	0.236637734125171	0.251093613298338	0.203644646924829
"MB54"	0.208281573498965	0.230861723446894	0.176980198019802
"MB55"	0.201853344077357	0.224043715846995	0.166733306677329

Table S5. Minimal set of genes, Related to Figure 5 (attached dataset). Minimal sets of altered genes associated with each one of the 38 medulloblastoma patients from (Forget *et al.*, 2018). The labels of the original subgroups (clusters) and the ones assigned after the optimization procedure are reported.

Table S6. Multilayer network enrichment analysis, Related to Figure 5 (attached dataset). The table reports those associations (edges) among the minimal sets of genes that are enriched in all the patients of a cluster and unique of each cluster (WNT, SHH, G3, G4, G3-G4) for a specific layer of the multilayer network (see Methods: “Multilayer network enrichment analysis”). Association IDs are grounded in databases (see Methods: “Data sources for the construction of the multilayer network”).

Transparent Methods

Multilayer network definition

A network (i.e. a graph or a *monoplex*) is defined as a tuple $G = (V, E)$, where V denotes the set of nodes (or vertices) in the network and $E \subseteq V \times V$ denotes the set of edges (or links) connecting them (Bollobás 1998). A graph composed of multiple networks, called layers, is referred to as a multilayer network. A multilayer network is defined as a quadruplet $M = (V_M, E_M, V, L)$, where V denotes the set of nodes in the multilayer network, L denotes the set of layers $l \in L$, $V_M \subseteq V \times L$ denotes the sets of nodes $v \in V$ contained in each layer, and $E_M \subseteq V_M \times V_M$ denotes the sets of edges connecting tuples of nodes and layers $(v, l), (v', l') \in V_M$ (Kivela et al. 2014) (**Figure S1**). In a multilayer network, an edge can be intra-layer, i.e. it connects nodes in the same layer ($l = l'$), or inter-layer, i.e. it connects nodes from different layers ($l \neq l'$). We built a multilayer network consisting of 5 layers and inter-layer edges imposed only between the same nodes, if any, on different layers.

Multilayer community detection

Communities in the multilayer network have been detected using MolTi software (Didier, Valdeolivas, and Baudot 2018; Didier, Brun, and Baudot 2015), which is available at https://github.com/gilles-didier/MolTi-DREAM. MolTi adapts the Louvain clustering algorithm with modularity maximization to multilayer networks. The Louvain algorithm for community detection consists of two recursive steps. In the first step, nodes are assigned to communities and then moved to others until no increase in modularity is observed. In the second step, the identified communities are aggregated so that a new graph is created and the entire process starts again and proceeds until convergence.

A community (c) is defined as a group of densely connected nodes in the different layers $l \in L$. The algorithm is parametrized to the resolution parameter γ: the higher the value of γ, the smaller the size of the detected multilayer communities. In MolTi, modularity of a multilayer network X is defined as

$$Multilayer\ modularity = \sum_{l} \frac{w^{(l)}}{2m^{(l)}} \sum_{\substack{\{i,j\} \\ i \neq j}} \left(X_{i,j}^{(l)} - \gamma \frac{S_i^{(l)} S_j^{(l)}}{2m^{(l)}} \right) \delta_{c_i,c_j}$$

where the first sum runs over all layers of the multilayer network and the second over all edges $\{i,j\}$ of each layer l. $X^{(l)}_{i,j}$ is the weight of the edge $\{i,j\}$ in a layer l; $S^{(l)}_i$ is the sum of the weights of all the edges involving vertex i in that layer; $m^{(l)}$ is the sum of the weights of all the edges of that layer; $\delta_{ci,cj}$ is equal to 1 if i and j belong to the same community ($c_i = c_j$) and to 0 otherwise; γ is the resolution parameter; $w^{(l)}$ is the user-defined weight associated to the layer l. In our calculations, $w^{(l)}$ and $X^{(l)}_{i,j}$ are both equal to 1, so that $m^{(l)}$ represents the total number of edges in l and $S^{(l)}_i$ and $S^{(l)}_j$ represent the degree of nodes i and j, respectively.

Data sources for the construction of the multilayer network

We created a multilayer network consisting of five layers in which nodes represent genes (Entrez identifiers), intra-layer edges represent different types of associations retrieved from publicly available knowledge bases and inter-layer edges exist between the same nodes in the different layers (**Figure S2**). All the data was downloaded on October 19, 2019, and it is available at https://github.com/cirillodavide/gene_multilayer_network.

Molecular associations. In this layer, two genes are connected if a physical or genetic association exists. Molecular associations between human genes were obtained from BioGRID, release 3.5.177. BioGRID (Oughtred et al. 2019) is a multi-species database of interactions, curated from high-throughput datasets and individual studies. Among other prominent primary databases, BioGRID shows the highest coverage for both interactions and proteins (Bajpai et al. 2019).

Drug-target associations. In this layer, two genes are connected if they are both targets of the same drug. Drug-target associations between human genes were obtained from KEGG BRITE “Target-based Classification of Compounds”, release br08310. KEGG BRITE (Kanehisa et al. 2019) is a manually curated database of functional hierarchies of various biological objects, such as Drug classifications. The Target-based Classification of Compounds consists of six categories

(Protein-coupled receptors, Nuclear receptors, Ion channels, Transportes, Enzymes, Others). One-to-one and unclassified gene-target associations were excluded.

Variant-disease associations. In this layer, two genes are connected if they are both reported to be associated with the same disease in genome-wide association studies (GWAS). Variant-disease associations between human genes were obtained from Monarch Disease Ontology (MonDO), released 2019-09-30. MonDO (Mungall et al. 2017) is a multi-species ontology generated by merging and harmonizing multiple disease resources (ORDO/Orphanet, DO, OMIM, MESH, etc.). In MonDO, gene-disease associations are inferred by integrating gene variants (SNPs, SNVs, QTLs, CNVs, among others) from significant GWAS hits. We retrieved MonDO entries with associated OMIM identifiers from the OWL file, filtering for evidence code ECO:0000220 (sequencing assay evidence) through the Monarch Solr search service.

Pathway associations. In this layer, two genes are connected if they are both annotated to the same pathway. Pathway associations between human genes were obtained from Reactome, release 70. Reactome (Fabregat et al. 2018) is a manually curated pathway database. Associations were retrieved from the lowest level pathway diagram of Reactome hierarchy. We found that all annotations are associated with IEA (inferred from electronic annotations) and TAS (traceable author statement) evidence codes.

Metabolic reaction associations. In this layer, two genes are connected if they are involved in metabolic reactions where product metabolites of one reaction are reactant metabolites of the other one. Metabolic reaction associations between human genes were obtained from Recon3D (Brunk et al. 2018) through BiGG Models (http://bigg.ucsd.edu), released 2019-09-12. Recon3D is the largest human metabolic network model. Superconnected metabolites (e.g. ATP, CO_2, H_2O) (Croes et al. 2006) were excluded.

Data sources of medulloblastoma genes

We aim to study the community structures of a multilayer network that contains medulloblastoma-associated genes. We selected genes for our study from two sources: (1) genes mentioned in scientific publications about medulloblastoma identified via text mining; (2) genes that are altered in medulloblastoma patients

based on two recent proteogenomic studies (Forget et al. 2018; Archer et al. 2018). The text mined data have been used as a proof-of-concept for the multilayer community structure analysis. The proteogenomic datasets have been used to identify the minimal sets of genes that characterize the medulloblastoma subgroups.

Text mined medulloblastoma genes. PubTator Central (PTC) (Wei et al. 2019) was used to retrieve gene mentions in abstracts of scientific publications indexed in PubMed with the MeSH term "medulloblastoma" (D008527) in February 2020 (see Resource Availability: "Data and Code Availability").

Medulloblastoma genes from proteogenomic data. Subgroups of 38 medulloblastoma patients (WNT, SHH, G3, G4) were retrieved from (Forget et al. 2018). While 35 patients present DNA methylation, RNA sequencing, proteomic and phosphoproteomic profiles, 3 patients (MB10, MB21, MB33) present only partial molecular information (the three lack RNA sequencing) and were used for validation. Gene methylation levels were mapped from CpG sites using the biomaRt package in R. When multiple CpG sites fell on a gene position, the median value was considered; when it fell on a region that is not annotated, the nearest gene was considered. Based on these pre-processed datasets (Forget et al. 2018), lists of genes, henceforth called "altered genes", were obtained by selecting the top 30% of the distribution of each data type. All the items of such lists were converted to Entrez identifiers, resulting in a total of 14039.6 altered genes per patient on average (see Resource Availability: "Data and Code Availability").

Subgroups of 45 medulloblastoma patients (WNT, SHHa, SHHb, G3a, G3b and G4) were retrieved from (Archer et al. 2018). While 39 patients present DNA acetylation, RNA sequencing, proteomics and phosphoproteomics profiles, 6 patients lack RNA sequencing information, including all 3 patients of the WNT subgroup. When multiple DNA acetylation measurements were linked to the same gene, the median value was considered. Altered genes were obtained with the same criterion as previously described and gene symbols converted to Entrez identifiers, resulting in a total of 11608.6 (SD= 2264.524) altered genes per patient on average.

Multilayer community structure analysis

We analyzed how the multilayer community structure varies within a range of modularity resolution (γ) where the most dramatic changes in size and composition

of the-communities are observed before both reach a plateau. We identified the endpoint of this range as the value where the average community size, as a function of the number of communities, establishes a plateau, i.e. where the first derivative equals zero with 0.05 margin of error (**Figure S3**). The endpoint was found at $\gamma=12$ (964 multilayer communities), indicating that $\gamma\in(0,12]$ is the range of interest for our study.

To compare the trajectories of each gene along the communities, we computed the pairwise Hamming distance (Hamming 1950) among the vectors of communities visited by each gene in the range $\gamma\in(0,12]$ with an interval of 0.5. We refer to these vectors as multilayer community trajectories. The higher the distance, the more times two genes belong to different communities within this range (**Figure 2**).

Identification of the minimal set of genes that define medulloblastoma subgroups

The biomedical goal of the study is to identify the minimal number of genes that recapitulate the four biomedically relevant medulloblastoma subgroups (WNT, SHH, G3, and G4) (Forget et al. 2018). Identifying a minimal set of genes is crucial for both the definition of diagnostic signatures and the research on disease mechanisms.

To achieve this goal, we performed a series of hierarchical clustering analyses (Ward's linkage method) where the similarity between two patients (A and B) was measured as the Jaccard index (J) of sets of altered genes selected using two parameters, θ and λ:

$$J(A_{\theta,\lambda}, B_{\theta,\lambda}) = \frac{A_{\theta,\lambda} \cap B_{\theta,\lambda}}{A_{\theta,\lambda} \cup B_{\theta,\lambda}}$$

The parameter θ defines the maximum Hamming distance allowed to include genes in the analysis, while the parameter λ defines the maximum number of them that must co-occur in the same communities along their trajectories. For dimensionality reduction purpose, small values of θ and λ guarantee a selection of genes with similar trajectories and in minimal numbers. For instance, with $\theta = 2$ and $\lambda = 4$, patient similarity is computed using sets of at most four genes that did not belong to

the same communities at most twice along their trajectories. For each of these clustering analyses, we identified the optimal number of clusters using the partitioning around medoids (PAM) algorithm (Kaufman and Rousseeuw 1987) (**Table S1**).

Based on this approach, we formulated an optimization procedure to systematically evaluate values of θ and λ to identify the ones that maximize the accuracy of recapitualiting patient stratification into the four medulloblastoma subgroups (WNT, SHH, Group 3, and Group 4). We defined accuracy as

$$Accuracy = \frac{TP + TN}{TP + TN + FP + FN}$$

where true positives (TP) are patients of the same subgroup who are clustered together, true negatives (TN) are patients of different subgroups who are not clustered together, false positives (FP) are patients of different subgroups who are clustered together, and false negatives (FN) are patients of the same subgroup who are not clustered together. The same optimization procedure can also be formulated to maximize the Matthews Correlation Coefficient (MCC), which is defined as

$$MCC = \frac{TP \times TN - FP \times FN}{\sqrt{(TP + FP)(TP + FN)(TN + FP)(TN + FN)}}$$

In both cases, the optimal parameters found are θ = 0 and λ = 6, corresponding to an accuracy of 94.94% (**Figure 4** and **Table S2**) and an MCC 87% (**Table S3**). The optimal number of clusters based on PAM is 5, suggesting the existence of subtle differences in few patients (see Results: “Medulloblastoma patient stratification through multilayer structure analysis”).

Multilayer network enrichment analysis

To detect overrepresented features (drugs, pathways, etc.) that characterize each cluster, we performed a network enrichment analysis test (NEAT) (Signorelli, Vinciotti, and Wit 2016) in each layer of the multilayer network. NEAT tests whether

the number of edges between two groups of nodes is significantly higher (over-enriched) than by chance, assuming a hypergeometric null distribution. In our analyses, the two groups of nodes are (a) the minimal set of genes of a patient that are present in a layer, and (b) the genes annotated to a certain feature of that layer (e.g., the genes annotated to a specific drug in the drug layer). In the specific case of the molecular interaction layer, the annotation feature consists of the neighborhood of each gene of the minimal set of a patient. Once we identify significant hits for each patient using a p-value cutoff of 0.01 (Benjamini-Hochberg correction for multiple testing), we select those features that are enriched in all the patients of a cluster and unique to each cluster (**Table S6**).

Computational resources

All calculations were performed using the R statistical environment, in particular the packages stats (hierarchical clustering), fpc (k-medoids clustering), pvclust (clustering significance by multiscale bootstrap resampling), sigclust2 (clustering significance by Monte Carlo procedure), and neat (network enrichment analysis). To ease the detection and analysis of the multilayer community trajectories, we developed the R package CmmD, which is openly available at https://github.com/ikernunezca/CmmD.

Supplemental References

Archer, Tenley C., Tobias Ehrenberger, Filip Mundt, Maxwell P. Gold, Karsten Krug, Clarence K. Mah, Elizabeth L. Mahoney, et al. 2018. “Proteomics, Post-Translational Modifications, and Integrative Analyses Reveal Molecular Heterogeneity within Medulloblastoma Subgroups.” *Cancer Cell* 34 (3): 396–410.e8.

Bajpai, Akhilesh Kumar, Sravanthi Davuluri, Kriti Tiwary, Sithalechumi Narayanan, Sailaja Oguru, Kavyashree Basavaraju, Deena Dayalan, Kavitha Thirumurugan, and Kshitish K. Acharya. 2019. “How Helpful Are the Protein-Protein Interaction Databases and Which Ones?” *Cold Spring Harbor Laboratory*. https://doi.org/10.1101/566372.

Bollobás, Béla. 1998. “Ramsey Theory.” *Modern Graph Theory*. https://doi.org/10.1007/978-1-4612-0619-4_6.

Brunk, Elizabeth, Swagatika Sahoo, Daniel C. Zielinski, Ali Altunkaya, Andreas Dräger, Nathan Mih, Francesco Gatto, et al. 2018. “Recon3D Enables a Three-Dimensional View of Gene Variation in Human Metabolism.” *Nature Biotechnology* 36 (3): 272–81.

Croes, Didier, Fabian Couche, Shoshana J. Wodak, and Jacques van Helden. 2006. “Inferring Meaningful Pathways in Weighted Metabolic Networks.” *Journal of Molecular Biology* 356 (1): 222–36.

Didier, Gilles, Christine Brun, and Anaïs Baudot. 2015. “Identifying Communities from Multiplex Biological Networks.” *PeerJ* 3 (December): e1525.

Didier, Gilles, Alberto Valdeolivas, and Anaïs Baudot. 2018. “Identifying Communities from Multiplex Biological Networks by Randomized Optimization of Modularity.” *F1000Research* 7 (July): 1042.

Fabregat, Antonio, Steven Jupe, Lisa Matthews, Konstantinos Sidiropoulos, Marc Gillespie, Phani Garapati, Robin Haw, et al. 2018. “The Reactome Pathway Knowledgebase.” *Nucleic Acids Research* 46 (D1): D649–55.

Forget, Antoine, Loredana Martignetti, Stéphanie Puget, Laurence Calzone, Sebastian Brabetz, Daniel Picard, Arnau Montagud, et al. 2018. “Aberrant ERBB4-SRC Signaling as a Hallmark of Group 4 Medulloblastoma Revealed by Integrative Phosphoproteomic Profiling.” *Cancer Cell* 34 (3): 379–95.e7.

Hamming, R. W. 1950. “Error Detecting and Error Correcting Codes.” *Bell System Technical Journal*. https://doi.org/10.1002/j.1538-7305.1950.tb00463.x.

Kanehisa, Minoru, Yoko Sato, Miho Furumichi, Kanae Morishima, and Mao Tanabe. 2019. “New Approach for Understanding Genome Variations in KEGG.” *Nucleic*

Acids Research 47 (D1): D590–95.

Kaufman, Leonard, and Peter Rousseeuw. 1987. *Clustering by Means of Medoids*. https://wis.kuleuven.be/stat/robust/papers/publications-1987/kaufmanrousseeuw-clusteringbymedoids-l1norm-1987.pdf

Kimes, Patrick K., Yufeng Liu, David Neil Hayes, and James Stephen Marron. 2017. “Statistical Significance for Hierarchical Clustering.” *Biometrics* 73 (3): 811–21.

Kivela, M., A. Arenas, M. Barthelemy, J. P. Gleeson, Y. Moreno, and M. A. Porter. 2014. “Multilayer Networks.” *Journal of Complex Networks* 2 (3): 203–71.

Mungall, Christopher J., Julie A. McMurry, Sebastian Köhler, James P. Balhoff, Charles Borromeo, Matthew Brush, Seth Carbon, et al. 2017. “The Monarch Initiative: An Integrative Data and Analytic Platform Connecting Phenotypes to Genotypes across Species.” *Nucleic Acids Research* 45 (D1): D712–22.

Oughtred, Rose, Chris Stark, Bobby-Joe Breitkreutz, Jennifer Rust, Lorrie Boucher, Christie Chang, Nadine Kolas, et al. 2019. “The BioGRID Interaction Database: 2019 Update.” *Nucleic Acids Research* 47 (D1): D529–41.

Wei, Chih-Hsuan, Alexis Allot, Robert Leaman, and Zhiyong Lu. 2019. “PubTator Central: Automated Concept Annotation for Biomedical Full Text Articles.” *Nucleic Acids Research* 47 (W1): W587–93.

Signorelli, Mirko, Veronica Vinciotti, and Ernst C. Wit. 2016. “NEAT: An Efficient Network Enrichment Analysis Test.” *BMC Bioinformatics* 17 (1): 352.

Suzuki, Ryota, and Hidetoshi Shimodaira. 2006. “Pvclust: An R Package for Assessing the Uncertainty in Hierarchical Clustering.” *Bioinformatics* 22 (12): 1540–42.
